# Disease Burden and Attributable Risk Factors of Ovarian Cancer From 1990 to 2017: Findings From the Global Burden of Disease Study 2017

**DOI:** 10.3389/fpubh.2021.619581

**Published:** 2021-09-17

**Authors:** Zhangjian Zhou, Xuan Wang, Xueting Ren, Linghui Zhou, Nan Wang, Huafeng Kang

**Affiliations:** ^1^Department of Oncology, The Second Affiliated Hospital of Xi'an Jiaotong University, Xi'an, China; ^2^Department of Surgical Oncology, The First Affiliated Hospital of Xi'an Jiaotong University, Xi'an, China; ^3^Bone Marrow Transplantation Center, The First Affiliated Hospital, School of Medicine, Zhejiang University, Hangzhou, China

**Keywords:** ovarian cancer, Global Burden of Disease (GBD), incidence, death, disability adjusted life-years

## Abstract

**Aim:** We aimed to estimate the disease burden and risk factors attributable to ovarian cancer, and epidemiological trends at global, regional, and national levels.

**Methods:** We described ovarian cancer data on incidence, mortality, and disability-adjusted life-years as well as age-standardized rates from 1990 to 2017 from the Global Health Data Exchange database. We also estimated the risk factors attributable to ovarian cancer deaths and disability-adjusted life-years. Measures were stratified by region, country, age, and socio-demographic index. The estimated annual percentage changes and age-standardized rates were calculated to evaluate temporal trends.

**Results:** Globally, ovarian cancer incident, death cases, and disability-adjusted life-years increased by 88.01, 84.20, and 78.00%, respectively. However, all the corresponding age-standardized rates showed downward trends with an estimated annual percentage change of −0.10 (−0.03 to 0.16), −0.33 (−0.38 to −0.27), and −0.38 (−0.32 to 0.25), respectively. South and East Asia and Western Europe carried the heaviest disease burden. The highest incidence, deaths, and disability-adjusted life-years were mainly in people aged 50–69 years from 1990 to 2017. High fasting plasma glucose level was the greatest contributor in age-standardized disability-adjusted life-years rate globally as well as in all socio-demographic index quintiles and most Global Disease Burden regions. Other important factors were high body mass index and occupational exposure to asbestos.

**Conclusion:** Our study provides valuable information on patterns and trends of disease burden and risk factors attributable to ovarian cancer across age, socio-demographic index, region, and country, which may help improve the rational allocation of health resources as well as inform health policies.

## Introduction

Ovarian cancer (OC) is the most common cancer in females worldwide and has a high mortality rate (1–3). Around 290,000 new OC cases (3.4% of all new cancer cases in females) have been diagnosed annually (3). Based on the latest global cancer statistics published in 2018, the age-standardized incidence rate (ASIR) and death rate (ASDR) of OC were 6.6 and 3.9 per 100,000 people, respectively. Epidemiological data from Saudi Arabia (4), China (5), and India (6) showed a remarkable OC burden with associated incidence and mortality. The Global Burden of Disease (GBD) studies provide global, regional, and country-specific epidemiological data of diseases and injuries showing the burden, distribution, and trends in different countries and regions (7–9). At present, there are no comprehensive and comparable assessments of incidence, mortality, disability, and epidemiological trends of OC at the global scale or in most regions.

Aside from family history, genetic factors, such as BRCA mutations as well as non-genetic factors, such as diabetes mellitus, high body mass index (BMI), tobacco, and alcohol use are the main risk factors for OC (10). Various single-institute studies have demonstrated the correlations between OC and these risk factors (11–13). Patients with risk factors such as diabetes mellitus are reportedly at a notably high risk for OC, and interventions such as metformin use dramatically reduce OC incidence (14). A systematic review suggested that OC risk was inversely associated with intake of black tea or calcium, and positively associated with intake of skim/low-fat milk or lactose (15). Such findings imply the necessity of comprehensive and comparable assessments of risk factors attributable to OC, which could then help in the development of prevention and treatment strategies.

In this study, we evaluated the burden and risk factors attributable to OC by location, social-development index (SDI), and age, providing valuable information on the distribution and trends of incident cases, deaths, disability-adjusted-life-years (DALYs), and risk factors, which could be beneficial to the improvement of health resources allocation and in the informed formulation of policies.

## Materials and Methods

### Data Acquisition

Annual data (inclusive dates: 1990–2017) on incidence, death, DALYs, and the corresponding age-standardized rates (ASRs) as well as risk factors attributable to OC were searched in the Global Health Data Exchange (GHDx) database (http://ghdx.healthdata.org/). All data are computed for direct inquiry and download through the GBD Results Tool. Details of methodology were described in the database help page and previous publications (16). These data were segmented by SDI quintiles, regions, countries, and territories. SDI reflects the degree of social development and correlates with total fertility, per capita income, and average years of education (17). All countries and territories were sorted into five quintiles based on SDI (http://ghdx.healthdata.org/record/ihme-data/gbd-2017-socio-demographic-index-sdi-1950%E2%80%932017). GBD regions are not actual geopolitical units; rather, these are groupings of countries created for analytical purposes alone (http://www.healthdata.org/sites/default/files/files/Data_viz/GBD_2017_Tools_Overview.pdf). Risk is defined as exposure, behavior, or other factors that are causally related to an increased (or decreased) probability of OC. If the probability decreased, the risk was considered a protective factor. In this GBD database, all of these risks were organized in four Levels, where Level 1 represents the overarching categories (behavioral, environmental and occupational, and metabolic) nested within Level 1 risks; Level 2 contains both single risks and risk clusters (such as the high fasting plasma glucose level); Level 3 contains the disaggregated single risks from within Level 2 risk clusters (such as low birthweight and short gestation); and Level 4 details risks with the most granular disaggregation, such as for specific occupational carcinogens, the subcomponents of child growth failure, and suboptimal breastfeeding (18). GBD risk hierarchy with levels were shown in the Supplementary Table 2 of Supplementary Appendix 1 in this paper (18). Data of all level risks were extracted to evaluate their variation tendency and effect on OC.

This study followed the Guidelines for Accurate and Transparent Health Estimates Reporting (GATHER) for cross-sectional studies (19).

### Statistical Analyses

ASRs were analyzed to compare the OC incidence and mortality trends between different cohorts. DALYs refer to the years lived with disability and years of life lost (20). Estimated annual percentage changes (EAPCs) indicate ASR trends during a defined period. The specific EAPC algorithm has been described in our previous works (7, 9, 21, 22).

World maps and graphs were generated to display the distribution and change trends of global, regional, and national disease burden and risk factors attributable to OC. All calculations and figures were performed and made using EXCEL 2013 (Microsoft Corporation) and R software (version 4.0.0) with “openxlsx,” “ggplot2,” “RColorBrewer,” “maptools,” and other packages.

## Results

### Global OC Incidence

The incident cases of OC increased from 152,090 (95% UI: 145,450–162,170) to 286,130 (95% UI: 278.08 to 295.31) globally, with a total increase of 88.01% from 1990 to 2017. The ASIR values demonstrated a downtrend (EAPC: −0.10, 95% UI: −0.03 to 0.16) (Table 1). In 2017, the prevalence case number of OC was 1,353,050. Most of these cases were distributed in East, South, and Southeast Asia (Supplementary Figure 1). In all SDI quintiles, the prevalence of OC in the high SDI quintile (410,127) ranked first, followed by the middle SDI quintile (335,709).

**Table 1:** The incidence of ovarian cancer, and its temporal trends from 1990 to 2017.

**Characteristics**	**1990**		**2017**		**1990–2017**	
	**Incident cases No. ×10^**3**^** **(95% UI)**	**ASIR per 100,000 No.** **(95% UI)**	**Incident cases No. ×10^**3**^** **(95% UI)**	**ASIR per 100,000 No.** **(95% UI)**	**Change in Incidence No.** **(%)**	**EAPC No.** **(95% CI)**
**Global**	152.09 (145.45–162.17)	6.71 (6.43–7.14)	286.13 (278.08–295.31)	6.83 (6.63–7.05)	88.01	−0.10 (−0.03 to 0.16)
**SDI**						
High SDI	74.91 (73.80–76.06)	11.28 (11.11–11.45)	91.49 (88.39–94.67)	9.20 (8.89–9.52)	22.13	−0.92 (−1.03 to −0.81)
High-middle SDI	34.04 (32.08–35.82)	6.31 (5.96–6.64)	59.26 (57.22–61.32)	6.38 (6.15–6.60)	74.08	−0.19 (−0.31 to 0.07)
Middle SDI	21.88 (20.50–24.62)	3.68 (3.46–4.13)	67.61 (64.75–70.72)	5.69 (5.46–5.95)	209.00	1.55 (1.48–1.62)
Low-middle SDI	14.17 (12.30–17.81)	4.17 (3.64–5.21)	47.69 (42.75–56.05)	6.74 (6.07–7.82)	236.56	1.81 (1.76–1.86)
Low SDI	6.76 (5.12–10.04)	3.49 (2.69–5.09)	19.20 (17.00–22.32)	4.58 (4.06–5.34)	184.02	0.91 (0.73–1.09)
**Regions**						
Andean Latin America	0.31 (0.27–0.35)	2.36 (2.10–2.65)	1.88 (1.64–2.16)	6.42 (5.60–7.37)	506.45	4.19 (3.38–4.19)
Australasia	1.31 (1.27–1.36)	10.69 (10.29–11.09)	1.89 (1.65–2.16)	8.37 (7.31–9.63)	44.27	−1.06 (−1.16 to −0.97)
Caribbean	0.30 (0.27–0.34)	2.03 (1.84–2.32)	1.67 (1.50–1.91)	6.34 (5.69–7.27)	456.67	4.18 (3.26–5.11)
Central Asia	1.42 (1.24–1.69)	4.85 (4.24–5.75)	2.83 (2.65–3.01)	6.16 (5.79–6.54)	99.30	0.95 (0.80–1.11)
Central Europe	8.66 (8.43–8.90)	11.02 (10.71–11.32)	10.87 (10.35–11.39)	11.06 (10.53–11.61)	25.52	−0.03 (−0.09 to 0.15)
Central Latin America	2.59 (2.51–2.66)	4.66 (4.53–4.79)	9.50 (9.02–9.99)	7.25 (6.89–7.62)	266.80	1.66 (1.47–1.85)
Central Sub-Saharan Africa	0.55 (0.39–0.74)	3.71 (2.76–4.73)	1.44 (1.11–1.83)	4.30 (3.31–5.43)	161.82	0.36 (0.21–0.50)
East Asia	16.19 (14.92–18.47)	2.92 (2.70–3.34)	43.75 (41.18–46.25)	4.22 (3.96–4.46)	170.23	1.18 (0.91–1.46)
Eastern Europe	16.06 (14.74–18.01)	10.00 (9.11–11.29)	17.39 (16.52–18.25)	9.84 (9.29–10.41)	8.21	−0.30 (−0.30 to 0.01)
Eastern Sub-Saharan Africa	3.16 (2.54–4.22)	6.56 (5.38–8.53)	7.02 (5.87–8.03)	6.72 (5.67–7.62)	122.15	−0.25 (−0.43 to −0.06)
High-income Asia Pacific	6.39 (6.26–6.52)	5.87 (5.74–5.99)	12.10 (11.34–12.80)	7.12 (6.65–7.60)	89.36	0.90 (0.62–1.19)
High-income North America	21.81 (21.33–22.40)	11.77 (11.52–12.11)	27.68 (26.20–29.12)	9.54 (8.96–10.10)	26.91	−1.09 (−1.26 to −0.92)
North Africa and Middle East	4.00 (3.41–5.36)	3.89 (3.36–5.14)	12.77 (12.01–13.63)	5.22 (4.91–5.55)	219.25	1.18 (1.11–1.25)
Oceania	0.09 (0.06–0.12)	4.34 (3.29–5.81)	0.31 (0.23–0.43)	7.05 (5.54–9.17)	244.44	1.99 (1.87–2.10)
South Asia	12.74 (10.91–16.48)	3.70 (3.19–4.79)	48.75 (43.90–56.10)	6.34 (5.76–7.20)	282.65	1.95 (1.79–2.12)
Southeast Asia	9.93 (8.49–12.83)	5.80 (5.01–7.42)	27.41 (24.13–31.86)	7.93 (7.00–9.19)	176.03	1.19 (1.11–1.27)
Southern Latin America	1.78 (1.64–1.92)	6.93 (6.38–7.48)	3.10 (2.75–3.51)	7.46 (6.62–8.48)	74.16	0.20 (0.02–0.38)
Southern Sub-Saharan Africa	0.91 (0.83–1.00)	4.97 (4.51–5.42)	2.10 (1.89–2.27)	6.04 (5.45–6.54)	130.77	0.75 (0.16–1.34)
Tropical Latin America	3.10 (2.97–3.25)	5.35 (5.15–5.60)	7.96 (7.60–8.32)	6.24 (5.96–6.53)	156.77	0.34 (0.18–0.49)
Western Europe	39.11 (38.40–39.86)	13.22 (12.96–13.49)	40.41 (38.33–42.59)	10.04 (9.49–10.56)	3.32	−1.22 (−1.31 to −1.14)
Western Sub-Saharan Africa	1.69 (1.34–2.32)	3.34 (2.66–4.59)	5.30 (4.18–6.69)	4.47 (3.54–5.59)	213.61	1.10 (1.09–1.12)

*ASIR, age standardized incidence rate; EAPC, estimated annual percentage change; CI, confidence interval; UI, uncertainty interval; SDI, socio-demographic index*.

**Figure 1:**
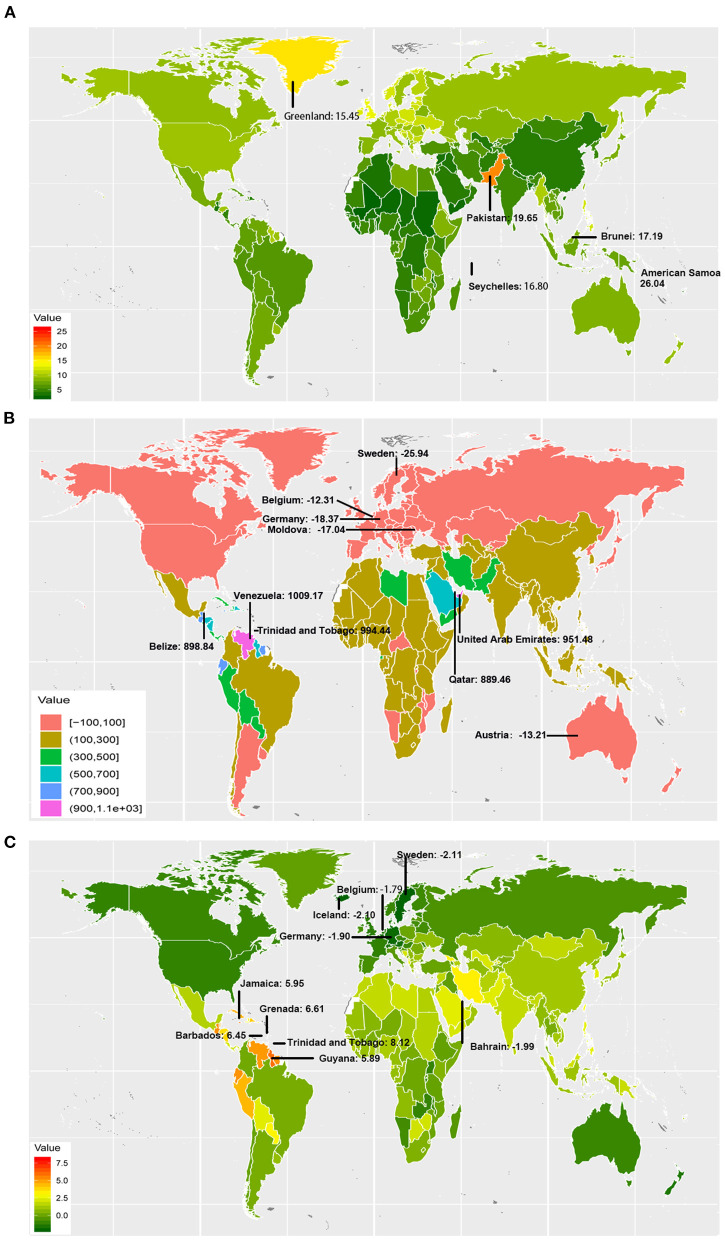
The incidence burden of ovarian cancer in 195 countries and territories. **(A)** The ASIR (per 100,000 people) of ovarian cancer globally in 2017; American Samoa, Pakistan, Brunei, Seychelles, and Greenland had the top 5 ASIR in 2017. **(B)** The relative change (%) in incident cases of ovarian cancer between 1990 and 2017; the greatest changes were exhibited in Venezuela and Sweden. **(C)** The EAPC of ovarian cancer ASIR from 1990 to 2017; the greatest EAPC were exhibited in Trinidad and Tobago, and Sweden. ASIR, age-standardized incidence rate; EAPC, estimated annual percentage change.

OC incident cases increased in a total of 184 countries and territories. The incident cases in Venezuela increased most from 106.25 to 1178.45 (Figure 1 and Supplementary Table 1). Compared with other countries and territories, higher number of incident cases were observed in China (40646.50, 95% UI: 38111.86–43093.19) and India (31441.05, 95% UI: 27725.81–36329.42), whereas fewer cases were observed in Kiribati (1.42, 95% UI: 0.96–1.95) and Northern Mariana Islands (1.89, 95% UI: 1.54–2.30) (Supplementary Figure 2 and Supplementary Tables 2, 3). On the other hand, American Samoa had the highest ASIR (26.04 per 100,000 people), whereas Mali had the lowest (2.33 per 100,000 people) in 2017 (Figure 1 and Supplementary Figure 3). The video shows the dynamic changes of countries with the top 10 ASIR of OC from 1990 to 2017 (Supplementary Video 1).

**Figure 2:**
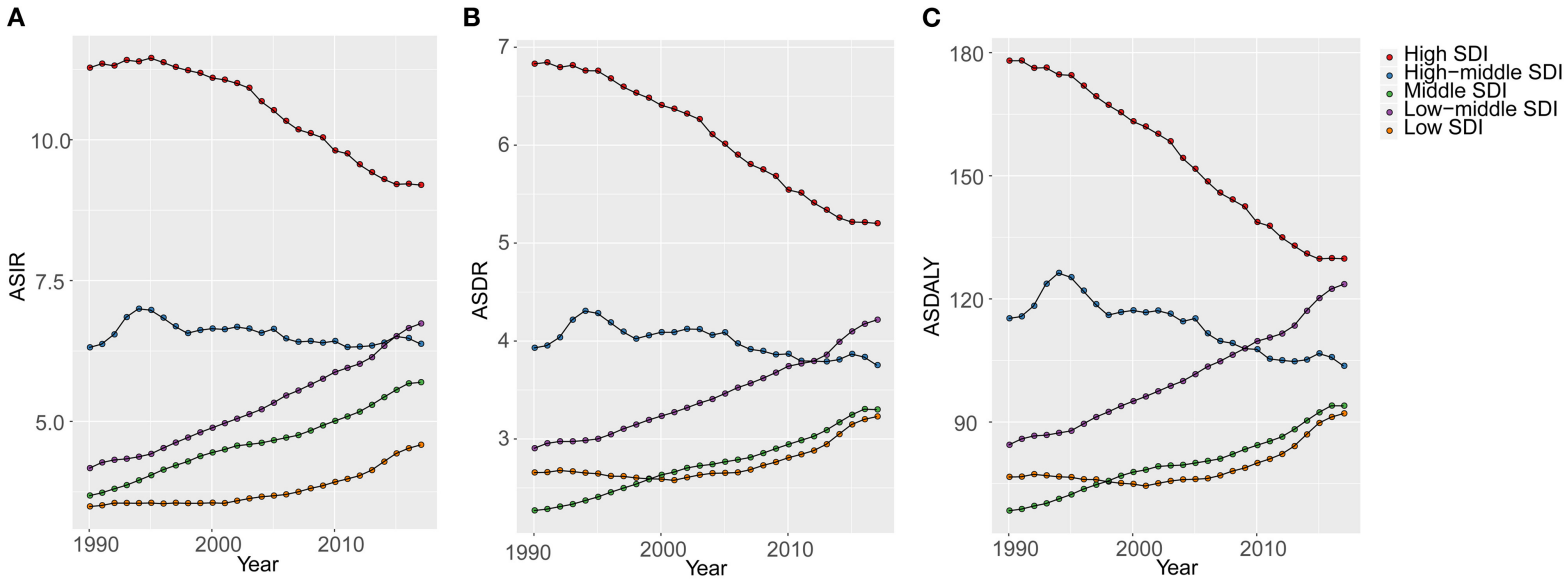
The changes of age-standardized rates of different SDI quintiles from 1990 to 2017 (per 100,000). **(A)** ASIR, age standardized incidence rate; **(B)** ASDR, age standardized death rate; **(C)** DALY, disability adjusted life-year. SDI, socio-demographic index; GBD, global burden of diseases.

OC incident cases increased in 16 GBD regions from 1990 to 2017 (Table 1). The greatest increase was observed in Andean Latin America (506.45%). In the high and high-middle SDI quintiles, incident cases increased whereas the corresponding ASIRs decreased (EAPC: −0.92 and −0.19, respectively) (Table 1). The highest (EAPC: 1.81) and lowest (EAPC: 0.91) increase in OC incidence were observed in the low-middle and low SDI quintiles, respectively. The ASIRs trended upwards in the low, low-middle, and middle SDI quintiles over 28 years from 1990 to 2017 (Figure 2). On the other hand, ASIRs demonstrated a temporary increase in 1995 and 1996 in the high-middle and high SDI quintiles, respectively.

**Figure 3:**
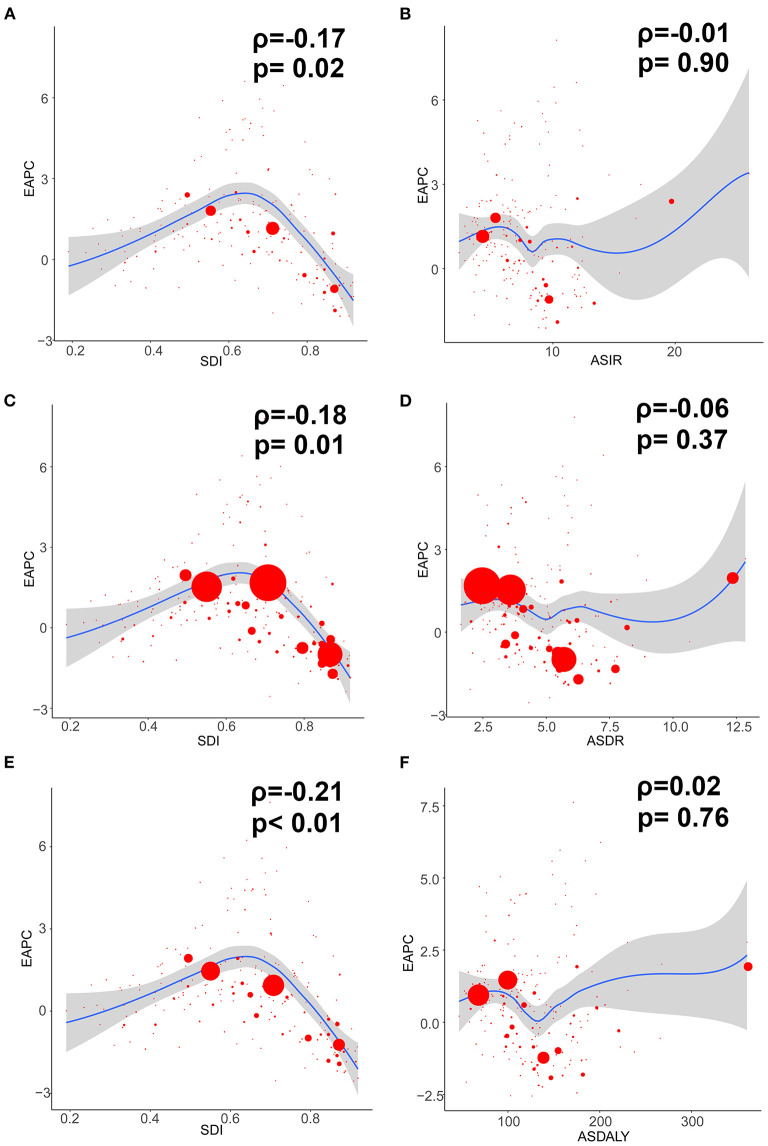
The correlation between EAPC and ovarian cancer ASR (per 100,000) in 1990 as well as SDI in 2017. The circles represent countries and territories that were available on SDI data. The size of circle is increased with the cases of ovarian cancer. The *R* and *P*-values presented were derived from Pearson correlation analysis. **(A)** EAPC and SDI in incidence; **(B)** EAPC and ASIR; **(C)** EAPC and SDI in death; **(D)** EAPC and ASDR; **(E)** EAPC and SDI in DALYs; **(F)** EAPC and age-standardized DALY rate. ASIR, age standardized incidence rate; ASDR, age standardized death rate; EAPC, estimated annual percentage change; SDI, socio-demographic index; DALY, disability adjusted life-year.

In addition, the EAPC of ASIR of 195 countries and territories was correlated with SDI (ρ = −0.169, 95% CI: −0.303 to −0.029, *P* = 0.018), but not correlated with ASIR itself (ρ = −0.009, 95% CI: −0.149 to 0.132, *P* = 0.905; Figures 3A,B).

### Global OC Mortality

The OC death cases increased globally by 84.20%, from 95,540 (95% UI: 91,780–101,190) to 175,980 (95% UI: 171,380–181,200) between 1990 and 2017. By contrast, the corresponding ASDR decreased worldwide (EAPC: −0.33, 95% UI: −0.38 to −0.27) (Table 2).

**Table 2:** The death of ovarian cancer, and its temporal trends from 1990 to 2017.

**Characteristics**	**1990**		**2017**		**1990–2017**	
	**Deaths No. ×10^**3**^** **(95% UI)**	**ASDR per 100,000 No.** **(95% UI)**	**Deaths No. ×10^**3**^** **(95% UI)**	**ASDR per 100,000 No.** **(95% UI)**	**Change in Death No.** **(%)**	**EAPC No.** **(95% CI)**
**Global**	95.54 (91.78–101.19)	4.36 (4.19–4.61)	175.98 (171.38–181.20)	4.14 (4.03–4.26)	84.20	−0.33 (−0.38 to −0.27)
**SDI**						
High SDI	49.14 (48.49–49.86)	6.83 (6.74–6.93)	60.86 (58.83–62.87)	5.20 (5.03–5.37)	23.85	−1.17 (−1.25 to −1.09)
High-middle SDI	20.88 (19.89–21.88)	3.93 (3.74–4.12)	36.37 (35.22–37.50)	3.75 (3.63–3.87)	74.19	−0.36 (−0.48 to −0.23)
Middle SDI	11.78 (11.27–13.39)	2.27 (2.14–2.53)	38.84 (37.31–40.48)	3.30 (3.17–3.44)	229.71	1.40 (1.35–1.45)
Low-middle SDI	8.77 (7.67–10.96)	2.90 (2.55–3.61)	27.25 (24.68–31.11)	4.22 (3.83–4.79)	210.72	1.40 (1.33–1.47)
Low SDI	4.59 (3.56–6.65)	2.66 (2.09–3.79)	12.13 (10.79–24.09)	3.23 (2.88–3.75)	164.27	0.65 (0.45–0.85)
**Regions**						
Andean Latin America	0.18 (0.16–0.21)	1.58 (1.41–1.75)	1.07 (0.94–1.22)	3.80 (3.33–4.31)	494.44	3.71 (2.95–4.48)
Australasia	0.85 (0.82–0.88)	6.58 (6.37–6.82)	1.26 (1.11–1.44)	4.99 (4.41–5.69)	48.24	−1.18 (−1.25 to −1.11)
Caribbean	0.18 (0.17–0.21)	1.30 (1.18–1.49)	1.04 (0.94–1.17)	3.86 (3.50–4.35)	477.78	4.01 (3.11–4.93)
Central Asia	0.86 (0.75–1.03)	3.03 (2.63–3.59)	1.65 (1.57–1.75)	3.78 (3.58–3.99)	91.86	0.93 (0.77–1.09)
Central Europe	5.70 (5.55–5.84)	6.83 (6.66–6.99)	7.78 (7.43–8.18)	6.89 (6.57–7.23)	36.49	0.10 (−0.02 to 0.22)
Central Latin America	1.47 (1.43–1.51)	3.05 (2.97–3.13)	5.20 (4.94–5.46)	4.09 (3.89–4.28)	253.74	1.11 (0.96–1.26)
Central Sub-Saharan Africa	0.38 (0.28–0.49)	2.87 (2.20–3.55)	0.94 (0.72–1.18)	3.20 (2.43–4.09)	147.37	0.26 (0.11–0.40)
East Asia	8.03 (7.42–9.23)	1.62 (1.49–1.86)	26.53 (25.04–28.06)	2.48 (2.35–2.63)	230.39	1.66 (1.45–1.87)
Eastern Europe	10.65 (9.91–11.73)	6.06 (5.63–6.70)	11.27 (10.85–11.72)	5.64 (5.42–5.87)	5.82	−0.51 (−0.83 to −0.20)
Eastern Sub-Saharan Africa	2.14 (1.76–2.78)	5.04 (4.18–6.41)	4.30 (3.62–4.88)	4.82 (4.08–5.46)	100.93	−0.46 (−0.64 to −0.29)
High-income Asia Pacific	3.80 (3.73–3.87)	3.36 (3.30–3.42)	6.55 (6.23–6.88)	3.12 (2.96–3.29)	72.37	−0.34 (−0.49 to −0.20)
High-income North America	13.98 (13.70–14.33)	6.97 (6.84–7.15)	18.62 (17.80–19.46)	5.67 (5.39–5.94)	33.19	−1.01 (−0.48 to −0.23)
North Africa and Middle East	2.47 (2.14–3.25)	2.69 (2.35–3.50)	6.81 (6.42–7.23)	3.10 (2.93–3.30)	175.71	0.59 (0.52–0.65)
Oceania	0.04 (0.03–0.06)	2.53 (1.98–3.23)	0.14 (0.11–0.18)	3.92 (3.29–4.85)	250.00	1.86 (1.76–1.96)
South Asia	8.29 (7.12–10.83)	2.72 (2.35–3.53)	29.55 (27.07–32.61)	4.15 (3.81–4.56)	256.45	1.48 (1.30–1.66)
Southeast Asia	5.17 (4.45–6.57)	3.45 (2.99–4.34)	13.56 (12.03–15.64)	4.09 (3.65–4.70)	162.28	0.68 (0.62–0.75)
Southern Latin America	1.27 (1.17–1.37)	4.86 (4.49–5.26)	2.03 (1.81–2.29)	4.54 (4.05–5.14)	59.84	−0.31 (−0.47 to 0.15)
Southern Sub-Saharan Africa	0.56 (0.50–0.62)	3.41 (2.98–3.77)	1.35 (1.22–1.46)	4.14 (3.75–4.49)	141.07	0.92 (0.46–1.39)
Tropical Latin America	1.86 (1.80–1.93)	3.58 (3.47–3.71)	4.78 (4.60–5.00)	3.73 (3.59–3.90)	156.99	−0.06 (−0.21 to 0.09)
Western Europe	26.48 (26.05–26.92)	7.99 (7.87–8.12)	28.33 (26.80–29.81)	5.86 (5.53–6.16)	6.99	−1.31 (−1.37 to −1.25)
Western Sub-Saharan Africa	1.18 (0.94–1.62)	2.58 (2.06–3.53)	3.22 (2.57–4.01)	3.19 (2.55–3.97)	172.88	0.79 (0.75–0.83)

*ASDR, age standardized death rate; EAPC, estimated annual percentage change; CI, confidence interval; UI, uncertainty interval; SDI, socio-demographic index*.

From 1990 to 2017, OC death cases decreased in 11 countries, and the corresponding ASDRs decreased in 60 countries (Supplementary Table 4). The highest number of death cases were observed in China (25040.26, 95% UI: 23558.50–26505.37) and India (20621.80, 95% UI: 18228.44–23704.20). The countries with the lowest number of death cases were Kiribati (0.76, 95% UI: 0.53–1.02) and Northern Mariana Islands (0.91, 95% UI: 0.77–1.09) (Supplementary Figure 2 and Supplementary Tables 2, 3). On the other hand, American Samoa had the highest ASDR (12.85 per 100,000 people), whereas Mali had the lowest (1.64 per 100,000 people) in 2017 (Supplementary Figures 3, 4). The video shows the dynamic changes of countries with the top 10 OC ASDRs from 1990 to 2017 (Supplementary Video 2).

OC death cases increased in all regions whereas the ASDRs decreased in 8 regions (Table 2). Compared with data from 1990, OC death cases in 2017 increased in all SDI quintiles. ASDRs decreased in the high and high-middle SDI quintiles (−1.17 and −0.36, respectively). The ASDRs trended upwards in the middle and low-middle SDI quintiles before 2017, and the opposite trend was observed in the high SDI quintiles after the rise and fall from 1990 to 1993 (Figure 2). Moreover, ASDRs demonstrated a decrease after two peaks in 1994 (4.31) and 2002–2003 (4.12) in the high-middle SDI quintiles, whereas ASDRs in the low SDI quintiles demonstrated an increase after a low peak in 2001 (2.58) (Figure 2).

Additionally, the EAPC of ASDR of 195 countries and territories was correlated with SDI (ρ = −0.182, 95% CI: −0.315 to −0.043, *P* = 0.011), but not correlated with ASDR itself (ρ = −0.065, 95% CI: −0.204 to 0.077, *P* = 0.371; Figures 3C,D).

### Global OC-Related DALYs

OC-related DALYs increased from 2,625,250 (95% UI: 2,493,770–2,829,950) to 4,673,030 (95% UI: 4,528,650–4,828,610) in the past 28 years. However, the DALY ASR decreased globally (EAPC: −0.38, 95% UI: −0.32 to −0.25) (Table 3).

**Table 3:** The DALYs of ovarian cancer, and its temporal trends from 1990 to 2017.

**Characteristics**	**1990**		**2017**		**1990–2017**	
	**DALYs No. ×10^**3**^** **(95% UI)**	**Age standardized DALY rate (per 100,000)** **(95% UI)**	**DALYs No. ×10^**3**^** **(95% UI)**	**Age standardized DALY rate (per 100,000)** **(95% UI)**	**Change in DALYs No.** **(%)**	**EAPC No.** **(95% CI)**
**Global**	2625.25 (2493.77–2829.95)	115.97 (110.29–124.73)	4673.03 (4528.65–4828.61)	110.94 (107.51–114.64)	78.00	−0.38 (−0.32 to −0.25)
**SDI**						
High SDI	1165.76 (1145.94–1185.90)	178.01 (175.02–181.12)	1286.70 (1243.45–1329.61)	129.78 (125.52–134.12)	10.37	−1.42 (−1.35 to −1.28)
High-middle SDI	619.01 (584.87–650.41)	115.22 (98.00–102.08)	986.76 (952.27–1019.77)	103.65 (99.98–107.15)	59.41	−0.63 (−0.76 to −0.50)
Middle SDI	398.06 (372.24–448.21)	68.37 (64.00–76.82)	1136.41 (1089.47–1187.13)	93.93 (90.10–98.14)	185.49	1.13 (1.07–1.20)
Low-middle SDI	285.87 (248.30–359.55)	84.40 (73.52–105.68)	865.01 (778.22–1001.88)	123.55 (111.45–142.26)	202.59	1.42 (1.37–1.48)
Low SDI	150.70 (114.44–223.63)	76.59 (58.92–111.58)	383.56 (339.68–445.46)	92.06 (81.68–106.87)	154.52	0.38 (0.58–0.78)
**Regions**						
Andean Latin America	6.17 (6.93–5.47)	48.10 (42.56–53.84)	31.36 (27.28–35.98)	108.38 (94.20–124.08)	408.27	3.45 (2.73–4.18)
Australasia	20.68 (19.97–21.49)	171.47 (165.44–178.21)	26.57 (23.38–30.47)	118.78 (104.23–136.21)	28.48	−1.53 (−1.60 to −1.46)
Caribbean	5.59 (4.99–6.57)	38.30 (34.20–44.87)	26.91 (25.57–32.90)	107.98 (96.55–124.64)	381.40	3.83 (2.97−4.70)
Central Asia	26.91 (23.54–31.76)	93.11 (81.37–110.03)	51.43 (48.38–54.70)	111.15 (104.61–118.13)	91.12	0.54 (0.72–0.89)
Central Europe	160.77 (156.34–164.85)	201.83 (196.37–206.75)	187.24 (178.37–196.50)	188.57 (179.79–197.81)	16.46	−0.22 (−0.32 to −0.12)
Central Latin America	47.50 (46.20–48.84)	88.15 (85.74–90.59)	155.32 (147.47–163.15)	118.61 (112.62–124.54)	227.00	1.16 (1.03–1.29)
Central Sub-Saharan Africa	12.39 (8.98–16.59)	80.77 (59.66.41–104.33)	29.85 (22.91–38.23)	88.14 (68.24–111.90)	140.92	0.15 (0.00–0.30)
East Asia	279.83 (257.75–319.49)	51.68 (47.72–59.14)	735.86 (692.70–779.50)	68.62 (64.57–72.74)	162.97	0.95 (0.71–1.18)
Eastern Europe	297.05 (274.68–329.81)	181.51 (167.37–202.60)	295.74 (283.21–309.03)	164.36 (157.04–172.69)	−0.44	−0.67 (−1.00 to −0.33)
Eastern Sub-Saharan Africa	70.94 (57.05–94.27)	146.17 (119.01–190.79)	141.10 (118.32–161.45)	137.51 (116.21–156.12)	98.90	−0.56 (−0.75 to −0.37)
High-income Asia Pacific	110.19 (107.97–112.44)	100.02 (98.00–102.08)	146.36 (138.57–155.16)	89.81 (84.81–95.70)	32.83	−0.42 (−0.56 to −0.28)
High-income North America	322.94 (315.58–332.21)	178.80 (174.68–184.13)	401.40 (380.99–421.83)	136.49 (128.84–144.04)	24.30	−1.27 (−1.40 to −1.13)
North Africa and Middle East	79.12 (67.42–106.24)	77.46 (66.35–103.03)	213.12 (200.76–226.90)	88.49 (83.42–93.94)	169.36	0.55 (0.49–0.61)
Oceania	1.51 (1.12–2.05)	78.05 (58.89–103.58)	5.10 (3.91–6.84)	116.84 (92.79–150.95)	237.75	1.63 (1.72–1.81)
South Asia	268.96 (230.29–350.55)	77.09 (66.23–100.47)	914.75 (831.99–1021.95)	119.33 (108.88–132.59)	240.11	1.54 (1.36–1.72)
Southeast Asia	172.75 (146.62–224.15)	103.43 (88.42–132.80)	423.82 (372.40–494.86)	121.20 (106.63–141.00)	145.34	0.63 (0.56–0.70)
Southern Latin America	33.43 (30.88–36.20)	130.72 (120.78–141.33)	50.53 (44.83–57.41)	121.97 (107.97–138.61)	51.15	−0.32 (−0.47 to −0.17)
Southern Sub-Saharan Africa	17.59 (15.89–19.26)	97.78 (87.95–106.83)	38.58 (35.00–41.98)	111.47 (101.11–121.05)	119.33	0.56 (−0.02 to 1.14)
Tropical Latin America	57.85 (55.67–60.30)	100.79 (97.30–104.77)	133.77 (128.31–139.95)	103.94 (99.71–108.70)	131.24	−0.05 (−0.25 to 0.01)
Western Europe	597.25 (586.96–608.43)	204.71 (201.13–208.51)	559.91 (528.82–590.19)	140.59 (133.04–148.17)	−6.25	−1.58 (−1.65 to −1.52)
Western Sub-Saharan Africa	35.84 (28.58–49.00)	71.19 (56.93–97.25)	102.69 (81.41–129.01)	87.76 (69.81–110.20)	186.52	0.34 (−0.04 to 0.85)

*DALY, disability adjusted life-year; EAPC, estimated annual percentage change; CI, confidence interval; UI, uncertainty interval; SDI, socio-demographic index*.

Decreases in OC-related DALYs were observed in 21 countries, and the DALY ASRs decreased in 66 countries as well (Supplementary Table 5). The OC-related DALYs of Trinidad and Tobago increased by ~9-fold. China (692806.34, 95% UI: 649947.79–734597.46) had the highest DALYs, followed by India (609130.85, 95% UI: 536743.45–702242.28). On the other hand, the DALYs were low in Kiribati (25.86, 95% UI: 17.66–35.50) and Northern Mariana Islands (27.48, 95% UI: 22.63–33.17) (Supplementary Figure 2 and Supplementary Tables 2, 3). Furthermore, Pakistan had the highest DALY ASR (360.24 per 100,000 people), whereas Mali had the lowest (46.92 per 100,000 people) in 2017 (Supplementary Figures 3, 5). The video shows the dynamic changes of countries with the top 10 OC DALY ASRs from 1990 to 2017 (Supplementary Video 3).

Compared with data from 1990, the OC-related DALYs in 2017 decreased only in Eastern and Western Europe (−0.44 and −6.25%, respectively) (Table 3). DALY ASR decreases were observed in 9 GBD regions, of which Western Europe had the greatest decrease (−1.58). Most of the OC-related DALYs were distributed in South (914,750) and East Asia (735,860) in 2017 (Table 3). DALY ASRs had the highest increase in the Caribbean (3.83), whereas the lowest increase was observed in Central Sub-Saharan Africa (0.15) (Table 3). OC-related DALYs increased in all SDI quintiles, whereas the OC DALY ASRs decreased in the high-middle and high SDI quintiles (EAPC: −0.63 and −1.42, respectively). Additionally, the EAPC of DALY ASRs of 195 countries and territories was correlated with SDI (ρ = −0.209, 95% CI: −0.340 to −0.070, *P* = 0.003), but not correlated with DALY ASRs itself (ρ = 0.022, 95% CI: −0.119 to 0.162, *P* = 0.760; Figures 3E,F).

### Age Distribution of OC Incidence, Deaths, and DALYs

From 1990 to 2017, global OC incidence and DALYs were mainly distributed in populations aged 50–69 years followed by those aged 15–49 years (Supplementary Figure 6). Although OC death cases occurred mainly in populations aged 50–69 years, the number of OC death cases is higher in those aged >70 years compared to those aged 15–49 years. Additionally, no huge difference was observed in OC incidence, deaths, and DALYs with respect to age between 1990 and 2017. However, the proportion of patients aged 50–69 years decreased and became the lowest population among the considered age groups around year 2000.

### Risk Factors for OC Burden

**Figure 4:**
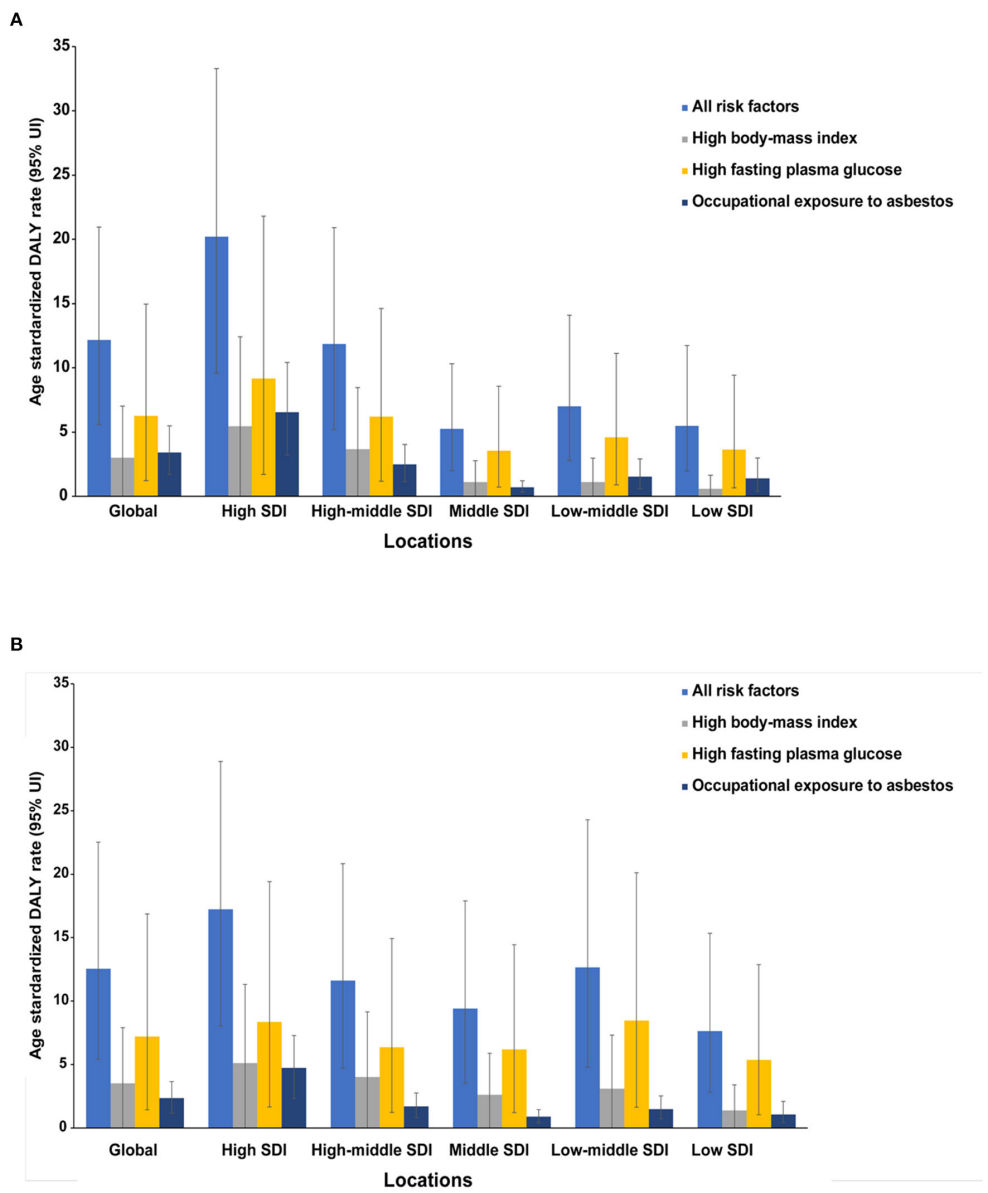
The ovarian cancer DALYs attributable to risk factors compared in 1990 **(A)** and 2017 **(B)** by SDI quintiles. DALY, disability-adjusted life year.

Global OC DALY ASRs attributable to all risks increased from 12.16 (95% UI: 5.59–20.95) to 12.56 (95% UI: 5.42–22.53) per 100,000 between 1990 and 2017 (Figure 4). Among the all risks, the high fasting plasma glucose level was the greatest contributor in DALY ASRs globally, as well as in all SDI quintiles in 1990 and 2017 (Figure 4). Another important contributor was high body mass index (BMI), which was the secondary contributor to DALY ASRs globally and in all SDI quintiles in 2017 (Figure 4). The third distributor in DALY ASRs in 2017 was the occupational exposure to asbestos, which contributed more to DALY ASRs globally and in most SDI quintiles in 1990.

**Figure 5:**
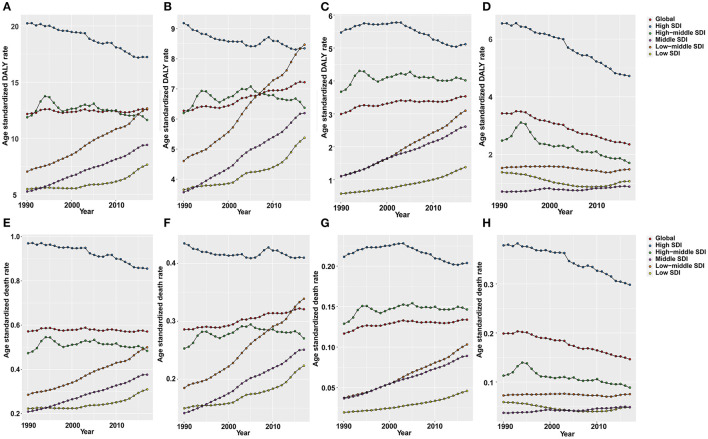
The age-standardized rates of DALYs and deaths (per 100,000) attributable to all risks **(A,E)**, high fasting plasma glucose **(B,F)**, high body mass index (BMI) **(C,G)**, and occupational exposure to asbestos **(D,H)** globally and in all SDI quintiles from 1990 to 2017.

As shown in Figure 5, OC DALY ASRs attributable to all risks increased mainly in the low, low-middle, and middle SDI quintiles, while it decreased in the high, high-middle SDI quintiles. Global OC DALY ASRs attributable to high fasting plasma glucose level maintained an upward trend from 1990 (6.27 per 100,000) to 2017 (7.22 per 100,000) but varied by SDI quintiles in 2017. Of the 534,059 (95% UI: 230,578–958,755) global OC DALYs attributable to all risks, 307,210 (95% UI: 61,032–717,907) was attributable to high fasting plasma glucose level. High fasting plasma glucose-related OC DALYs in 2017 increased globally when compared with data in 1990 (data not shown). Moreover, high fasting plasma glucose-related OC DALY ASRs increased in the low, low middle, and middle SDI quintiles, whereas in the high, high-middle SDI quintiles (Figure 5B), the values decreased.

Global OC DALY ASRs attributable to high BMI increased in 2017 (3.53 per 100,000) when compared with data in 1990 (2.99 per 100,000). The proportion of high BMI-attributed DALY ASRs decreased in the high SDI quintile, but increased in the other quintiles, especially in the middle and low-middle SDI quintiles (Figure 5C).

Global OC DALY ASRs attributable to occupational exposure to asbestos was 2.35 per 100,000 in 2017, which was a great decrease compared with data in 1990 (3.42 per 100,000) globally. The attributed DALY ASRs in the high and high-middle SDI quintiles decreased from 1990 to 2017, but were still higher than those in the other three SDI quintiles. Meanwhile, the attributed DALY ASRs showed a decrease in low and low-middle SDI quintiles and a increase in the middle SDI quintiles from 1990 to 2017 (Figure 5D).

The OC ASDRs attributable to these risk factors displayed patterns similar to the DALY ASRs (Figures 5E–H). While, OC ASDRs attributable to all risks decreased slightly from 0.571 per 100,000 in 1990 to 0.570 per 100,000 in 2017. In a comparison of data between 1990 and 2017, OC ASDRs attributable to high fasting plasma glucose level and high BMI increased, whereas OC ASDRs attributable to occupational exposure to asbestos decreased.

## Discussion

Our analysis showed the latest global patterns and trends on disease burden and risk factors attributable to OC. The incidence, deaths, and DALYs increased globally from 1990 to 2017 and has been in a continuous upward trend (Supplementary Figure 7). The corresponding ASRs of OC all showed general downward trends with a peak and trough around 1994 and 2014 (Supplementary Figure 8). Previous paper about the incidence and mortality burden of ovarian cancer confirmed our findings (23). Besides, the latest research about the global disease burden of women cancer revealed that the incidence, deaths, and DALYs of ovarian cancer were 294,420, 198,410, and 5,359,740, respectively in 2019 globally which showed increases from 2017 (24). These findings could be helpful to the allocation of the limited health service resources as well as the evaluation of interventions or programs. According to a global cancer burden study (from 1990 to 2016) (25), increases in OC incidence were mainly attributable to population growth (12.4%) and changes in age structure (14.9%) over the last decade, whereas the reverse contributed a 3.2% decrease. Specifically, in the high, high-middle, and middle SDI quintiles, changes in age structure were the main factor that contributed to changes in incidence (13.1, 14.6, and 20%, respectively). Furthermore, negative changes in ASIRs in the high (−9.4%) and high-middle (−8.4%) SDI quintiles were attributable to decreases in incidence. Population growth was the greatest contributor in the incidence changes in the low-middle (16.6%) and low SDI (32.3%) quintiles. Generally, population growth and aging are continuing and may still be the main factors contributing to the increase in OC incidence. China, India, the United States, Pakistan, and the Russian Federation were the top 5 countries with the highest incidence, mortality, and DALYs in not only 2017 but also a few years prior (26). European and North American regions had the highest ASRs in recent years, as described in published reports (10, 26, 27). Therefore, in the coming decades, the number of OC patients who need specialist treatment will also continue to increase.

High fasting plasma glucose level is the most important risk factor for OC deaths and DALYs globally. A previous case-control study in China showed that fasting plasma glucose and high BMI were significantly prevalent in OC (28). By searching the GHDx database, we also found that in China, DALYs and mortality attributable to high fasting plasma glucose levels decreased in 2017 after a long-term upward trend in 1990 to 2016 (data not shown). Moreover, various studies from other institutes have also reported the high risk for OC in patients with diabetes mellitus (29, 30). Mechanistic studies indicate that glucose in diabetes mellitus patients provides energy not only to normal cells but also to tumor cells, hence promoting tumor growth (31). Additionally, hyperinsulinemia caused by insulin resistance could promote cancer cell mitosis through molecules such as insulin receptor-A and insulin-like growth factor-1, or through activation of the insulin-like growth factor-1 receptor signaling pathway (32–34). Other studies have demonstrated that diabetes mellitus also promoted carcinogenesis through regulation of programmed cell death and immune system surveillance (35, 36). Moreover, some studies have investigated the effect of anti-diabetes medications or other treatments on OC risk. Several studies report that metformin and breastfeeding reduce OC risk in diabetes mellitus patients (14, 37, 38).

Although asbestos use is banned in most countries, there are millions of people still working in factories with asbestos exposure, and at least 90,000 people die from asbestos-related diseases or cancer diseases every year (39). Based on mortality data from the World Health Organization Health Statistics database for the year 2009, Argentina, Brazil, Colombia, and Mexico reflected the greatest numbers of estimated OC deaths attributable to occupational asbestos exposure in 5 years (40). Asbestos use has decreased for many years; nevertheless, governments should increase efforts to limit asbestos production and use and look for alternatives to reduce asbestos exposure. Occupational disease screening and routine physical examination of workers could also help improve the early detection rate of diseases. The association between high BMI and OC risk has been addressed in various studies (28, 41, 42). Obesity has always been a health topic of great concern. It is associated with many diseases, and people should control obesity through a reasonable diet, healthy work and rest, and exercise.

Studies of global cancer incidence, mortality, and DALYs based on the GHDx program provide high-quality estimates of cancer burden. However, these studies rely on the quality of actual raw data, which are unavailable in the GBD database. More accurate estimates of disease burden could be easily obtained in developed countries because they have timely and accurate disease registration data. Nevertheless, these estimates may be influenced by data reliability and poor enrollment rate biases, which could manifest in mathematical modeling errors. Moreover, heterogeneity in data acquisition and processing, including disease detection, diagnosis, and coding, may lead to results deviations. Our research is no exception to these limitations.

## Conclusion

OC disease burden increased worldwide, and the heaviest burden was distributed in South and East Asia and Western Europe. High fasting plasma glucose level was the greatest contributor in DALY ASRs globally. Our study provides valuable information on the patterns and trends of disease burden and risk factors attributable to OC across age, SDI, regions, and countries, which may help improve the rational allocation of health resources as well as inform policy formulation.

## Data Availability Statement

The original contributions presented in the study are included in the article/Supplementary Material, further inquiries can be directed to the corresponding author/s.

## Ethics Statement

The Ethics Committee of the Second Affiliated Hospital of Xi'an Jiaotong University determined that this study did not need ethical approval because it used publicly available data.

## Author Contributions

HK designed the research. ZZ, XW, and NW collected and arranged the data. ZZ, LZ, and NW performed the statistical analysis and made the figures and tables. ZZ, XR, XW, and HK wrote and revised the manuscript. All authors read, critically reviewed, and approved the final manuscript.

## Funding

This study was supported by Basic Research Program of Natural Science Foundation of Shaanxi Province (No. 2021JQ-422).

## Conflict of Interest

The authors declare that the research was conducted in the absence of any commercial or financial relationships that could be construed as a potential conflict of interest.

## Publisher's Note

All claims expressed in this article are solely those of the authors and do not necessarily represent those of their affiliated organizations, or those of the publisher, the editors and the reviewers. Any product that may be evaluated in this article, or claim that may be made by its manufacturer, is not guaranteed or endorsed by the publisher.

## Supplementary Material

Supplementary Figure 1 The crude prevalence cases (×1,000) of ovarian cancer in different GBD regions and SDI quintiles in 2017.

Supplementary Figure 2 The incidence, mortality and DALYs cases (×1,000) of ovarian cancer in 195 countries and territories in 2017. DALY, disability adjusted life-year.

Supplementary Figure 3 The countries with top 10 age-standardized rates (per 100,000 people) in 2017. **(A)** ASIR, age standardized incidence rate; **(B)** ASDR, age standardized death rate; **(C)** age-standardized DALY rate. DALY, disability adjusted life-year.

Supplementary Figure 4 The global death burden of ovarian cancer in 195 countries and territories in 2017. **(A)** The age-standardized death rate (per 100,000 people) of ovarian cancer in 2017; **(B)** the relative change in death cases between 1990 and 2017; **(C)** the EAPC of ovarian cancer ASDR. EAPC, estimated annual percentage change.

Supplementary Figure 5 The global DALY burden of ovarian cancer in 195 countries and territories in 2017. **(A)** The age-standardized DALY rate (per 100,000 people) of ovarian cancer in 2017; **(B)** the relative change in DALYs between 1990 and 2017; **(C)** the EAPC of the age-standardized DALY rate. ASDR, age-standardized death rate; EAPC, estimated annual percentage change.

Supplementary Figure 6 The proportion of different age groups in ovarian cancer by years. **(A)** Incidence. **(B)** Death. **(C)** DALY disability adjusted life-year.

Supplementary Figure 7 The global change in ovarian cancer incidence, death and DALYs from 1990 to 2017. DALY, disability-adjusted life year.

Supplementary Figure 8 The global change in age-standardized incidence, death, and DALYs rates of ovarian cancer from 1990 to 2017. DALY, disability-adjusted life year.

Supplementary Table 1 The incidence of ovarian cancer among 195 countries and territories, and its temporal trends from 1990 to 2017.

Supplementary Table 2 The top five countries and territories of ovarian cancer incidence, death, or DALYs in 2017.

Supplementary Table 3 The bottom five countries and territories of ovarian cancer incidence, death, or DALYs in 2017.

Supplementary Table 4 The deaths of ovarian cancer among 195 countries and territories, and their temporal trends from 1990 to 2017.

Supplementary Table 5 The DALYs of ovarian cancer among 195 countries and territories, and their temporal trends from 1990 to 2017.

Supplementary Video 1 The dynamic changes of countries with top 10 ASIR (per 100,000) of OC from 1990 to 2017 (MP4). This video shows the dynamic change of the top 10 countries with highest ASIR of OC annually from 1990 to 2017.

Supplementary Video 2 The dynamic changes of countries with top 10 ASDR (per 100,000) of OC from 1990 to 2017 (MP4). This video shows the dynamic change of the top 10 countries with highest ASDR of OC annually from 1990 to 2017.

Supplementary Video 3 The dynamic changes of countries with top 10 DALY ASR (per 100,000) of OC from 1990 to 2017 (MP4). This video shows the dynamic change of the top 10 countries with highest DALY ASR of OC annually from 1990 to 2017.
