# Supplementary figures and images for: Disease Burden and Attributable Risk Factors of Ovarian Cancer From 1990 to 2017: Findings From the Global Burden of Disease Study 2017
Source: Front Public Health. 2021 Sep 17;9:619581. doi: 10.3389/fpubh.2021.619581 (PMC8484795; doi:10.3389/fpubh.2021.619581)

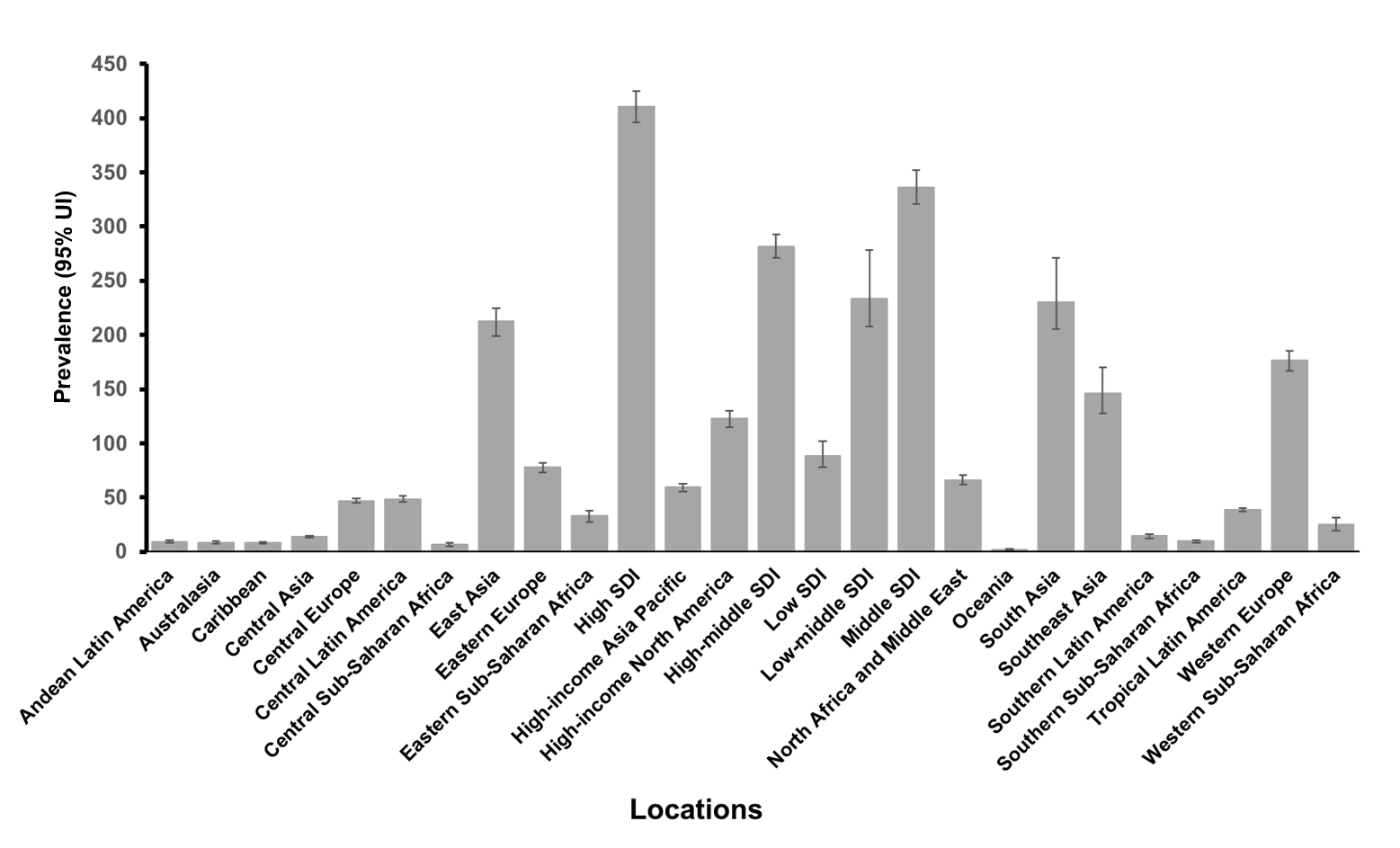

Supplement: Supplementary Figure 1 — The crude prevalence cases (×1,000) of ovarian cancer in different GBD regions and SDI quintiles in 2017. [file Image_1.TIF]

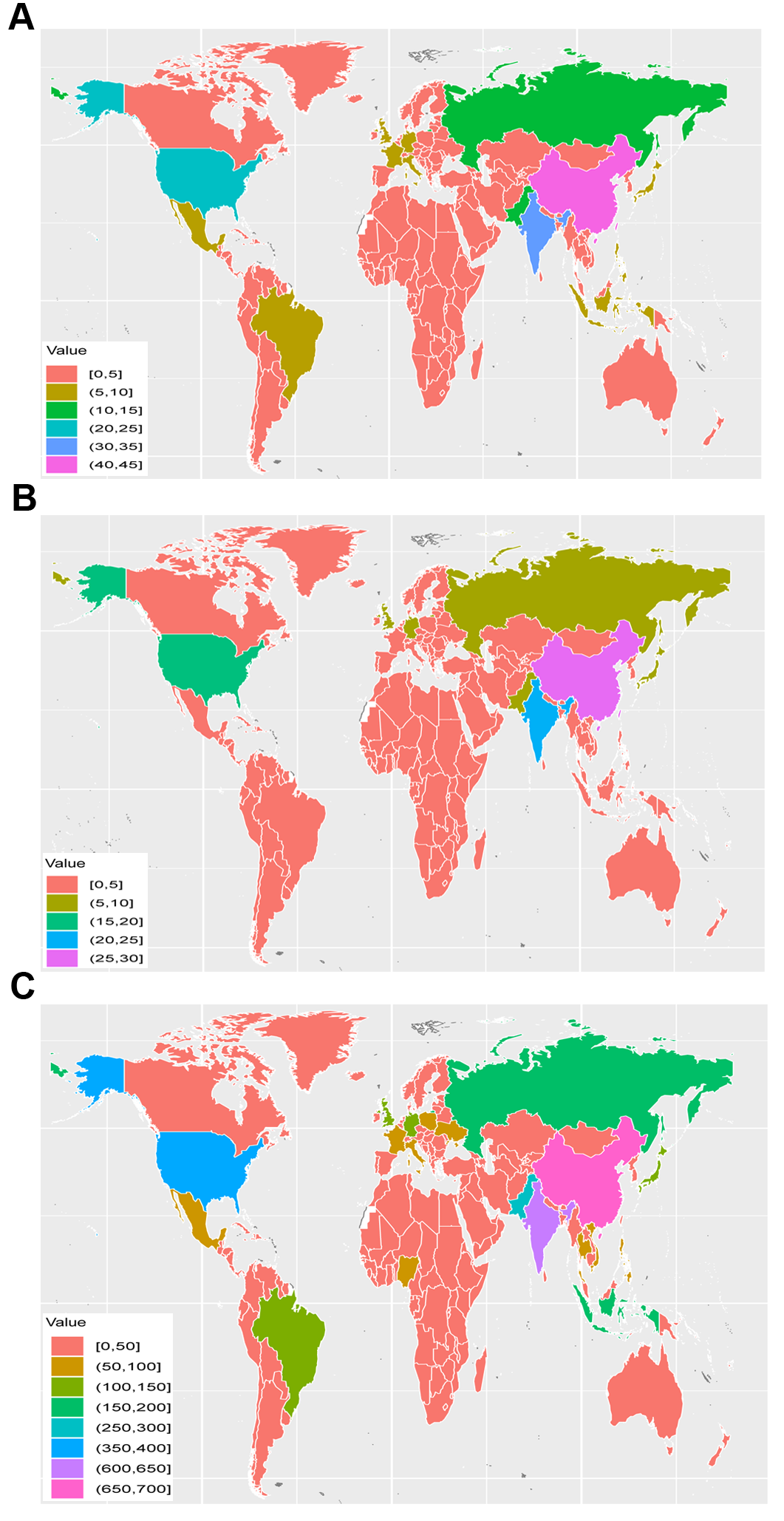

Supplement: Supplementary Figure 2 — The incidence, mortality and DALYs cases (×1,000) of ovarian cancer in 195 countries and territories in 2017. DALY, disability adjusted life-year. [file Image_2.TIF]

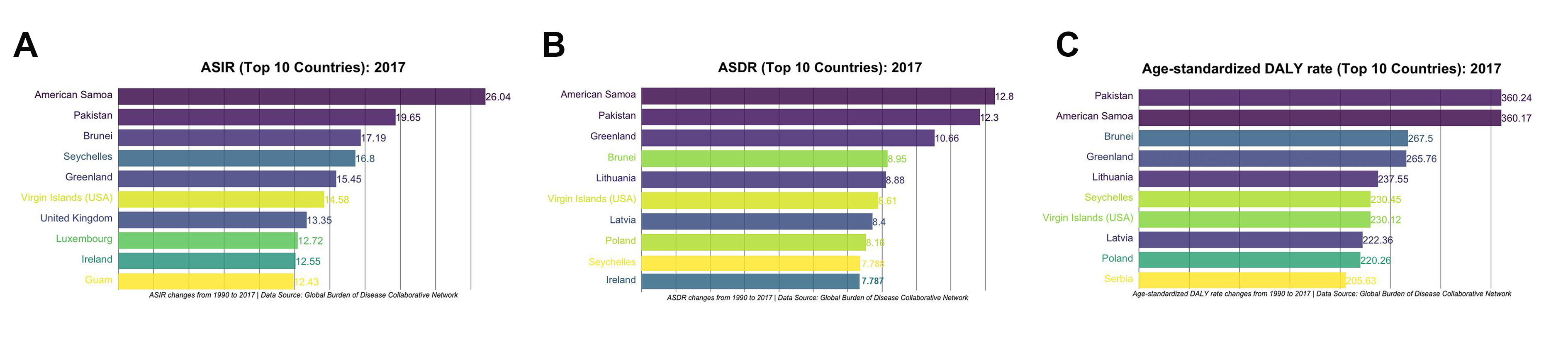

Supplement: Supplementary Figure 3 — The countries with top 10 age-standardized rates (per 100,000 people) in 2017. (A) ASIR, age standardized incidence rate; (B) ASDR, age standardized death rate; (C) age-standardized DALY rate. DALY, disability adjusted life-year. [file Image_3.TIF]

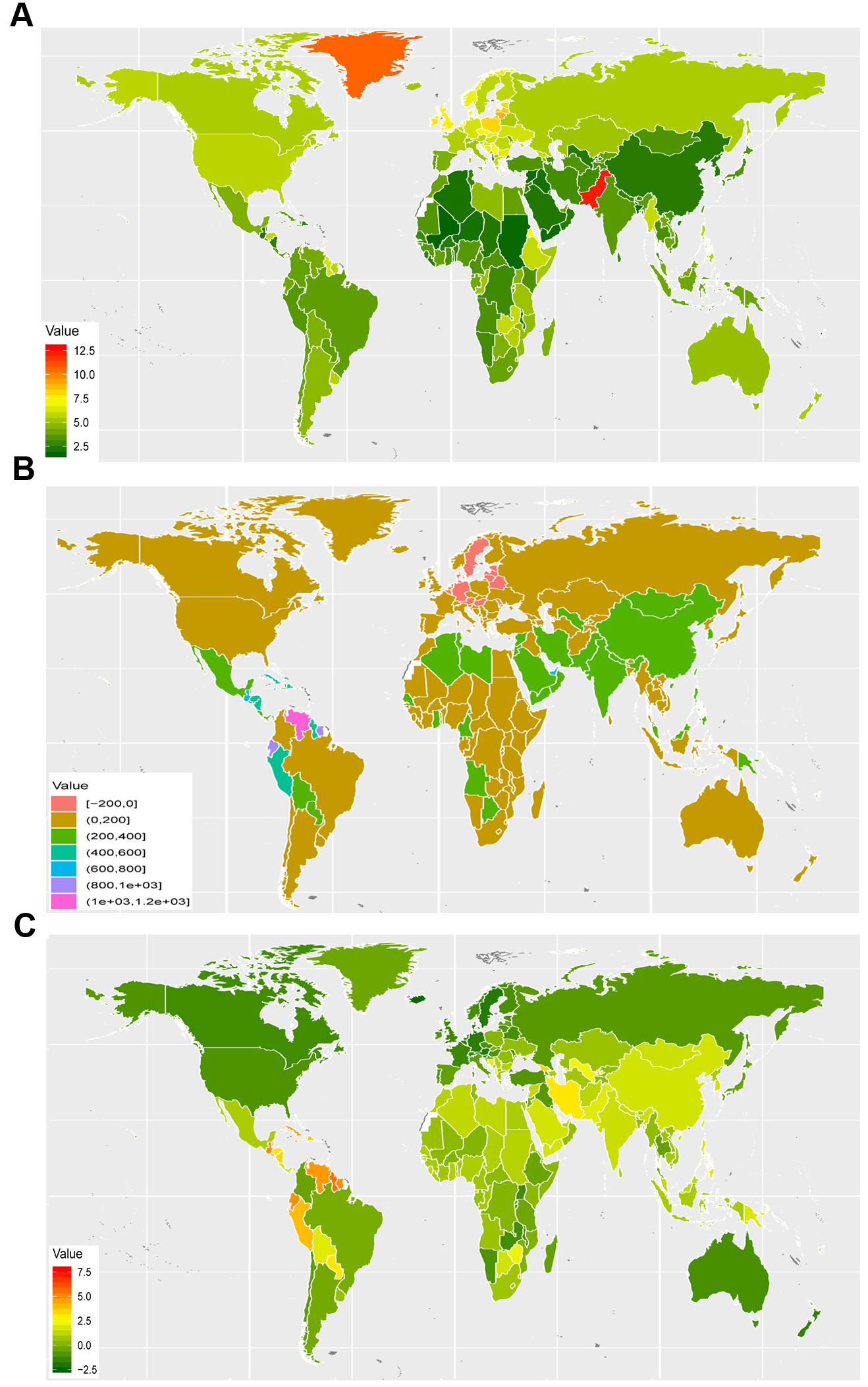

Supplement: Supplementary Figure 4 — The global death burden of ovarian cancer in 195 countries and territories in 2017. (A) The age-standardized death rate (per 100,000 people) of ovarian cancer in 2017; (B) the relative change in death cases between 1990 and 2017; (C) the EAPC of ovarian cancer ASDR. EAPC, estimated annual percentage change. [file Image_4.TIF]

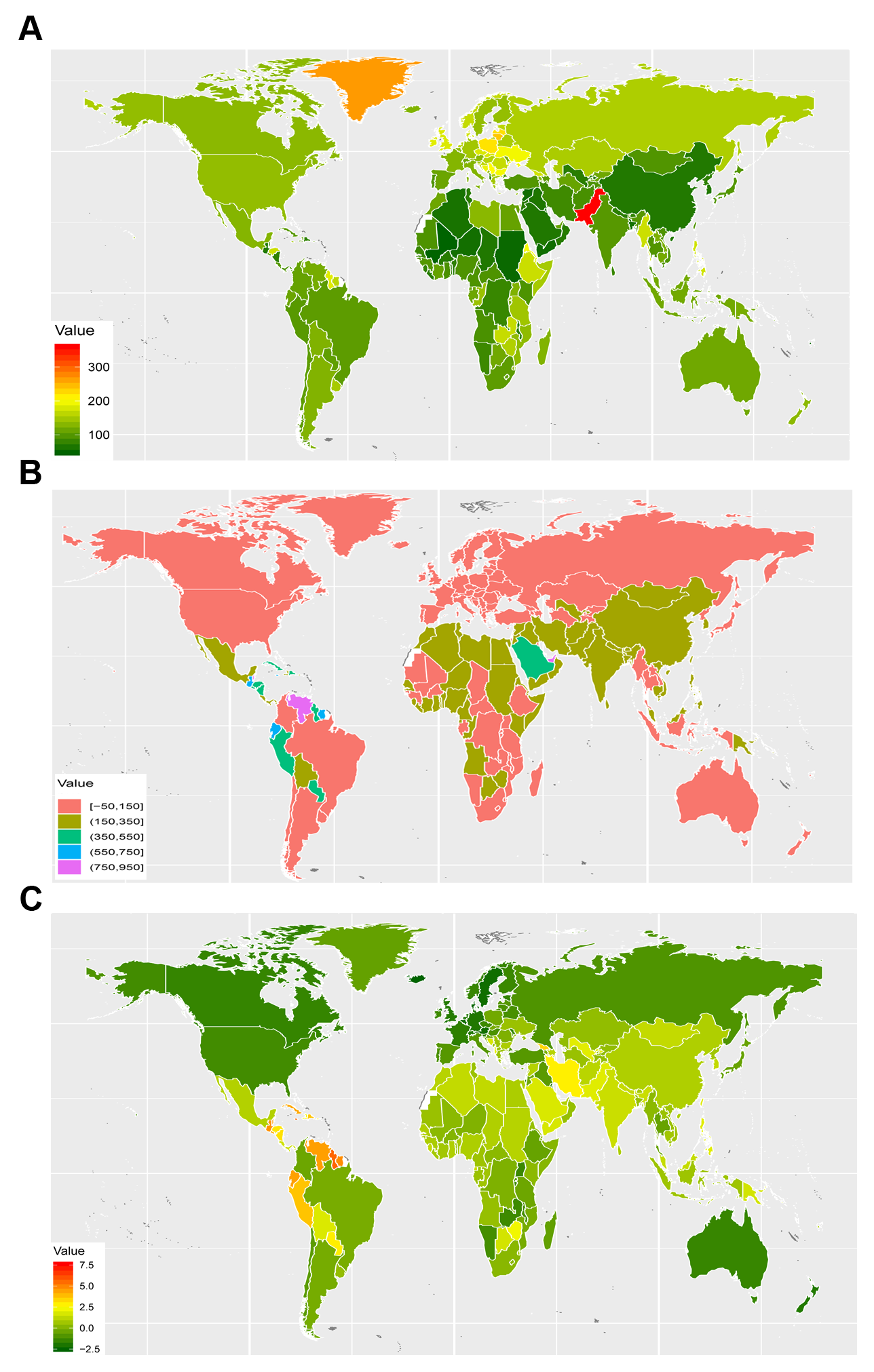

Supplement: Supplementary Figure 5 — The global DALY burden of ovarian cancer in 195 countries and territories in 2017. (A) The age-standardized DALY rate (per 100,000 people) of ovarian cancer in 2017; (B) the relative change in DALYs between 1990 and 2017; (C) the EAPC of the age-standardized DALY rate. ASDR, age-standardized death rate; EAPC, estimated annual percentage change. [file Image_5.TIF]

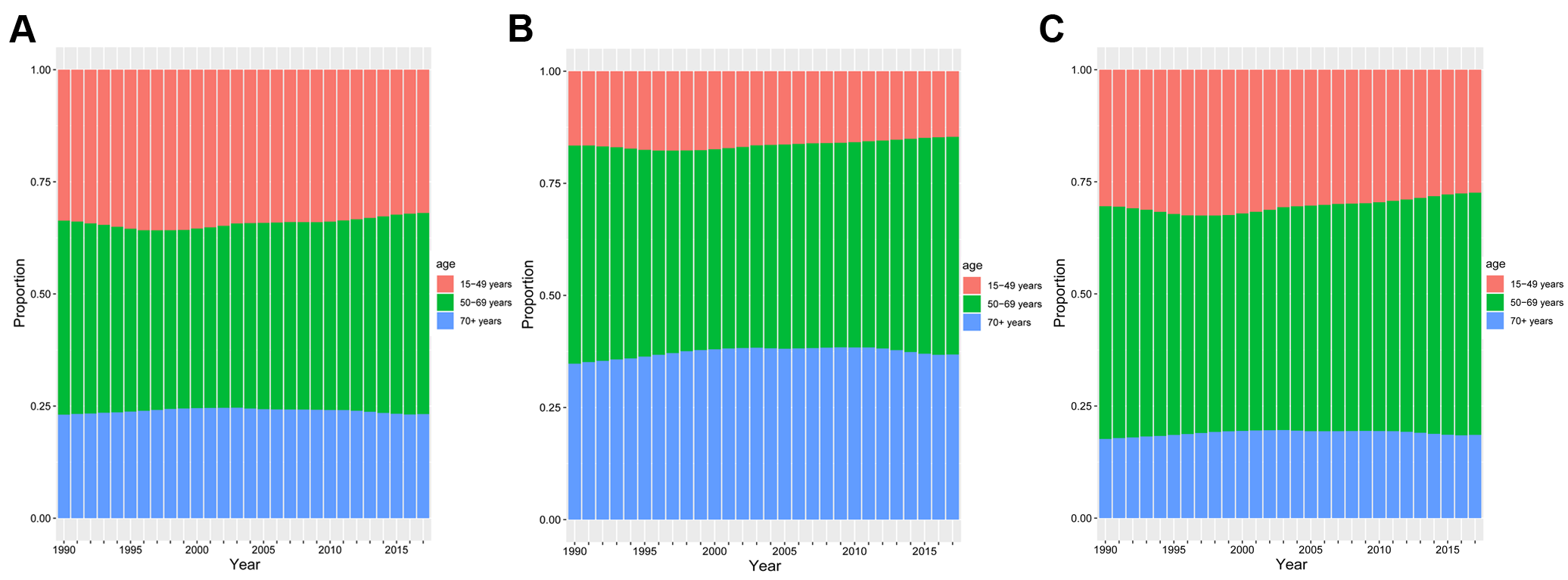

Supplement: Supplementary Figure 6 — The proportion of different age groups in ovarian cancer by years. (A) Incidence. (B) Death. (C) DALY disability adjusted life-year. [file Image_6.TIF]

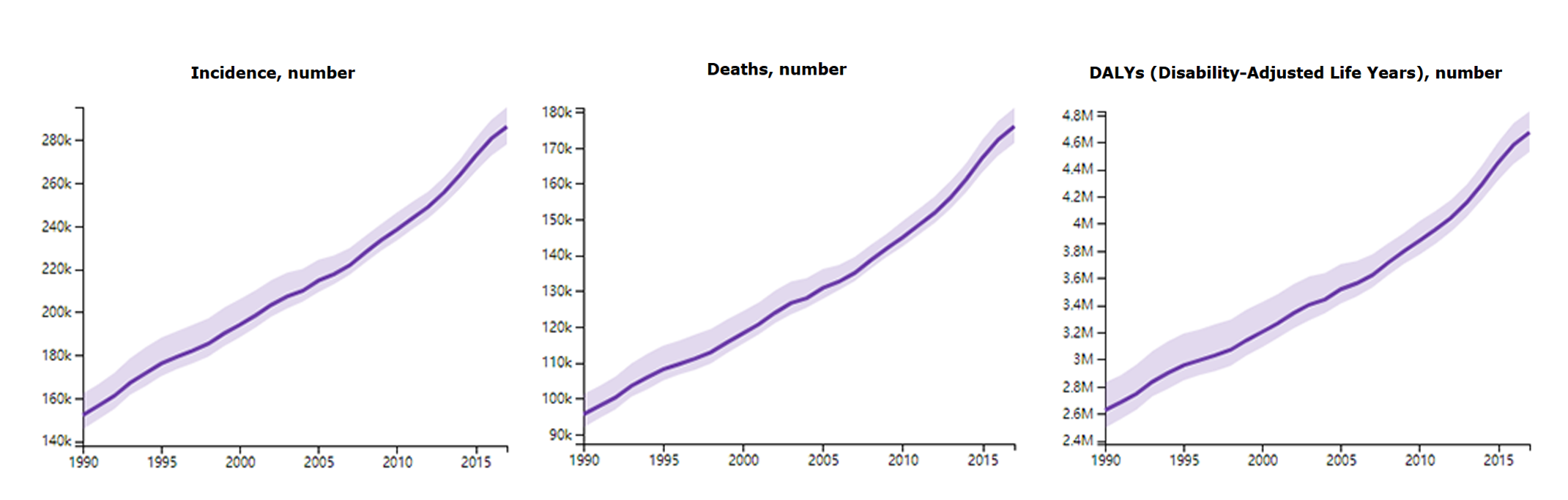

Supplement: Supplementary Figure 7 — The global change in ovarian cancer incidence, death and DALYs from 1990 to 2017. DALY, disability-adjusted life year. [file Image_7.TIF]

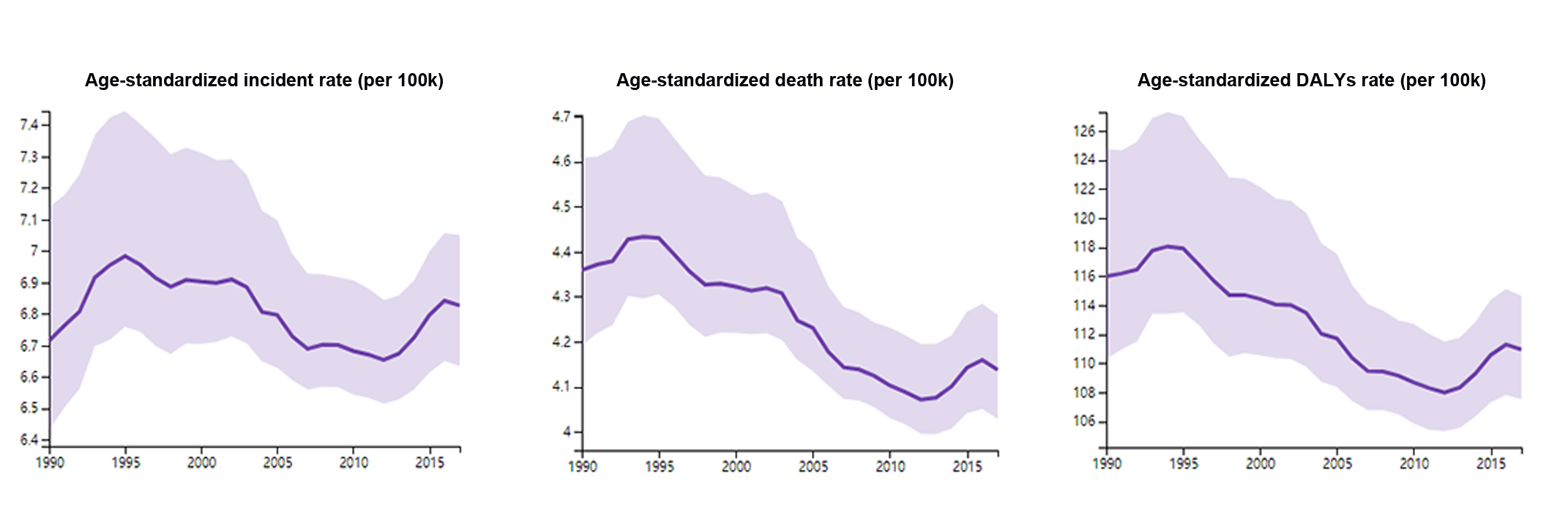

Supplement: Supplementary Figure 8 — The global change in age-standardized incidence, death, and DALYs rates of ovarian cancer from 1990 to 2017. DALY, disability-adjusted life year. [file Image_8.TIF]
