# Supplementary material for: Disease Burden and Attributable Risk Factors of Ovarian Cancer From 1990 to 2017: Findings From the Global Burden of Disease Study 2017
Source: Front Public Health. 2021 Sep 17;9:619581. doi: 10.3389/fpubh.2021.619581 (PMC8484795; doi:10.3389/fpubh.2021.619581)
Supplement: Supplementary Table 1 — The incidence of ovarian cancer among 195 countries and territories, and its temporal trends from 1990 to 2017. [file Table_1.DOCX]

**Supplementary Table 1 The incidence of ovarian cancer among 195 countries and territories, and its temporal trends from 1990 to 2017.**

| **Countries and territories** | **1990** | | **2017** | | **1990 - 2017** | |
| --- | --- | --- | --- | --- | --- | --- |
|  | **Incident cases No.** | **ASIR per 100,000** | **Incident cases No.** | **ASIR per 100,000 No.** | **Change in Incidence Number (%)** | **EAPC No. (95% CI)** |
| Afghanistan | 110.85 | 3.15 | 352.05 | 4.40 | 217.6 | 1.47 |
| Albania | 41.79 | 3.17 | 83.82 | 4.59 | 100.59 | 2.39 |
| Algeria | 177.49 | 2.35 | 664.97 | 3.57 | 274.66 | 1.81 |
| American Samoa | 1.79 | 12.13 | 6.29 | 26.04 | 251.71 | 3.37 |
| Andorra | 3.50 | 12.05 | 7.30 | 11.61 | 108.73 | -0.30 |
| Angola | 94.21 | 3.65 | 346.32 | 4.57 | 267.6 | 0.76 |
| Antigua and Barbuda | 0.59 | 2.05 | 5.01 | 9.15 | 755.13 | 5.51 |
| Argentina | 1245.15 | 7.03 | 2067.80 | 7.63 | 66.07 | 0.27 |
| Armenia | 115.90 | 7.17 | 161.26 | 7.37 | 39.14 | 0.17 |
| Australia | 1050.05 | 10.24 | 1562.02 | 8.19 | 48.76 | -0.96 |
| Austria | 903.33 | 14.44 | 784.02 | 9.44 | -13.21 | -1.74 |
| Azerbaijan | 136.40 | 4.21 | 350.25 | 5.97 | 156.78 | 1.38 |
| Bahrain | 8.00 | 8.06 | 29.96 | 6.43 | 274.67 | -1.99 |
| Bangladesh | 688.60 | 2.57 | 2179.08 | 3.17 | 216.45 | 1.05 |
| Barbados | 3.03 | 2.01 | 22.35 | 10.09 | 638.38 | 6.45 |
| Belarus | 750.67 | 9.97 | 682.42 | 8.37 | -9.09 | -0.76 |
| Belgium | 1109.81 | 14.02 | 973.17 | 9.18 | -12.31 | -1.79 |
| Belize | 0.69 | 1.28 | 6.88 | 4.34 | 898.84 | 4.82 |
| Benin | 34.32 | 2.80 | 115.67 | 3.77 | 237.05 | 1.04 |
| Bermuda | 3.86 | 10.83 | 5.26 | 8.90 | 36.47 | -0.85 |
| Bhutan | 4.52 | 3.00 | 14.96 | 4.30 | 230.83 | 1.36 |
| Bolivia | 73.88 | 3.58 | 341.29 | 6.90 | 361.96 | 2.40 |
| Bosnia and Herzegovina | 159.16 | 6.39 | 308.02 | 11.02 | 93.53 | 2.11 |
| Botswana | 17.15 | 4.43 | 58.92 | 6.48 | 243.68 | 2.04 |
| Brazil | 3064.51 | 5.42 | 7765.08 | 6.24 | 153.39 | 0.29 |
| Brunei | 6.55 | 9.73 | 35.00 | 17.19 | 434.06 | 2.99 |
| Bulgaria | 513.40 | 8.32 | 700.32 | 11.45 | 36.41 | 1.05 |
| Burkina Faso | 84.84 | 3.07 | 208.31 | 3.50 | 145.54 | 0.34 |
| Burundi | 95.57 | 6.45 | 139.25 | 5.39 | 45.7 | -0.97 |
| Cambodia | 169.25 | 5.11 | 513.79 | 6.94 | 203.57 | 1.17 |
| Cameroon | 102.86 | 3.62 | 349.01 | 4.67 | 239.31 | 0.84 |
| Canada | 1903.24 | 11.04 | 2782.01 | 9.07 | 46.17 | -0.97 |
| Cape Verde | 2.92 | 2.21 | 9.90 | 3.97 | 239.34 | 2.32 |
| Central African Republic | 29.87 | 3.94 | 57.32 | 4.29 | 91.88 | 0.28 |
| Chad | 39.36 | 2.36 | 100.14 | 3.17 | 154.4 | 1.15 |
| Chile | 391.82 | 6.59 | 798.30 | 6.73 | 103.74 | -0.06 |
| China | 15068.19 | 2.87 | 40646.50 | 4.12 | 169.75 | 1.14 |
| Colombia | 743.01 | 6.46 | 1933.25 | 6.82 | 160.19 | 0.18 |
| Comoros | 9.96 | 7.70 | 24.68 | 8.82 | 147.73 | 0.43 |
| Congo | 36.31 | 5.18 | 111.74 | 7.02 | 207.73 | 0.95 |
| Costa Rica | 35.17 | 3.33 | 168.56 | 6.32 | 379.33 | 2.15 |
| Cote d'Ivoire | 106.49 | 4.09 | 334.37 | 5.23 | 214 | 0.89 |
| Croatia | 506.12 | 14.42 | 492.35 | 12.13 | -2.72 | -0.23 |
| Cuba | 103.55 | 1.90 | 553.97 | 6.46 | 434.99 | 4.60 |
| Cyprus | 39.50 | 8.94 | 83.08 | 9.16 | 110.35 | 0.40 |
| Czech Republic | 1054.13 | 14.48 | 1146.31 | 11.92 | 8.74 | -0.98 |
| Democratic Republic of the Congo | 372.57 | 3.57 | 868.61 | 3.95 | 133.14 | 0.11 |
| Denmark | 633.08 | 16.05 | 650.56 | 12.33 | 2.76 | -1.38 |
| Djibouti | 8.20 | 7.97 | 31.43 | 9.12 | 283.24 | 0.35 |
| Dominica | 0.47 | 1.32 | 2.36 | 5.75 | 403.02 | 5.61 |
| Dominican Republic | 34.44 | 1.43 | 219.29 | 4.39 | 536.81 | 4.07 |
| Ecuador | 59.40 | 1.73 | 549.55 | 6.83 | 825.2 | 5.18 |
| Egypt | 724.70 | 4.16 | 2263.06 | 6.28 | 212.28 | 1.45 |
| El Salvador | 38.51 | 2.04 | 213.11 | 6.46 | 453.42 | 4.72 |
| Equatorial Guinea | 4.51 | 3.56 | 20.42 | 5.90 | 352.87 | 2.26 |
| Eritrea | 47.16 | 6.97 | 157.14 | 9.40 | 233.2 | 0.68 |
| Estonia | 167.80 | 13.77 | 162.29 | 11.81 | -3.28 | -0.92 |
| Ethiopia | 1026.98 | 8.13 | 2137.34 | 8.32 | 108.12 | -0.12 |
| Federated States of Micronesia | 1.47 | 5.02 | 3.66 | 8.66 | 149.26 | 2.12 |
| Fiji | 6.19 | 2.44 | 14.55 | 3.38 | 135.16 | 1.24 |
| Finland | 523.66 | 13.57 | 586.95 | 10.80 | 12.09 | -0.89 |
| France | 4920.06 | 11.45 | 5459.22 | 8.74 | 10.96 | -1.14 |
| Gabon | 15.20 | 4.57 | 36.43 | 5.82 | 139.69 | 0.85 |
| Georgia | 168.19 | 4.88 | 274.74 | 9.62 | 63.36 | 3.30 |
| Germany | 10879.28 | 15.76 | 8880.99 | 10.33 | -18.37 | -1.90 |
| Ghana | 141.69 | 3.35 | 513.14 | 4.81 | 262.16 | 1.28 |
| Greece | 548.38 | 7.43 | 960.63 | 9.94 | 75.18 | 0.98 |
| Greenland | 3.26 | 16.40 | 5.09 | 15.45 | 56.33 | -0.15 |
| Grenada | 0.96 | 2.55 | 7.92 | 11.92 | 725.29 | 6.61 |
| Guam | 2.30 | 4.60 | 11.00 | 12.43 | 379.36 | 4.36 |
| Guatemala | 28.23 | 1.13 | 268.70 | 3.84 | 851.96 | 5.26 |
| Guinea | 68.78 | 3.59 | 153.94 | 4.73 | 123.83 | 1.08 |
| Guinea-Bissau | 8.59 | 3.27 | 21.52 | 4.39 | 150.54 | 1.15 |
| Guyana | 5.66 | 2.18 | 36.90 | 10.24 | 552.06 | 5.89 |
| Haiti | 55.68 | 2.75 | 237.14 | 5.41 | 325.89 | 2.62 |
| Honduras | 50.49 | 3.83 | 357.23 | 9.72 | 607.48 | 3.68 |
| Hungary | 950.01 | 12.26 | 879.65 | 9.53 | -7.41 | -0.90 |
| Iceland | 20.78 | 14.48 | 21.57 | 8.84 | 3.81 | -2.10 |
| India | 8492.23 | 3.06 | 31441.05 | 5.20 | 270.23 | 1.80 |
| Indonesia | 3707.07 | 5.49 | 9268.60 | 7.28 | 150.02 | 1.01 |
| Iran | 467.72 | 2.78 | 2170.58 | 5.38 | 364.08 | 3.33 |
| Iraq | 179.83 | 3.69 | 575.97 | 3.70 | 220.28 | -0.22 |
| Ireland | 301.88 | 14.72 | 438.53 | 12.55 | 45.26 | -0.67 |
| Israel | 283.81 | 11.18 | 473.34 | 8.61 | 66.78 | -1.32 |
| Italy | 4476.27 | 10.30 | 5413.12 | 9.19 | 20.93 | -0.38 |
| Jamaica | 16.11 | 1.70 | 132.83 | 8.78 | 724.62 | 5.95 |
| Japan | 5768.86 | 6.64 | 9783.47 | 8.07 | 69.59 | 0.96 |
| Jordan | 36.42 | 3.92 | 163.91 | 4.57 | 350.07 | 0.28 |
| Kazakhstan | 553.06 | 6.80 | 874.37 | 8.57 | 58.1 | 0.64 |
| Kenya | 211.57 | 3.97 | 675.42 | 4.79 | 219.24 | 0.50 |
| Kiribati | 0.55 | 2.15 | 1.42 | 2.98 | 156.82 | 1.27 |
| Kuwait | 26.41 | 6.32 | 59.81 | 3.58 | 126.48 | -1.65 |
| Kyrgyzstan | 110.79 | 5.99 | 175.83 | 6.15 | 58.7 | 0.50 |
| Laos | 74.21 | 5.48 | 202.06 | 7.28 | 172.29 | 1.03 |
| Latvia | 268.03 | 12.79 | 246.03 | 12.01 | -8.21 | -0.37 |
| Lebanon | 123.84 | 8.77 | 451.23 | 12.42 | 264.35 | 1.13 |
| Lesotho | 23.97 | 3.99 | 51.48 | 6.69 | 114.8 | 2.44 |
| Liberia | 16.39 | 2.81 | 42.80 | 3.49 | 161.13 | 0.87 |
| Libya | 48.68 | 4.65 | 227.76 | 7.97 | 367.85 | 2.00 |
| Lithuania | 368.78 | 14.29 | 328.02 | 11.76 | -11.05 | -0.76 |
| Luxembourg | 44.86 | 15.37 | 58.40 | 12.72 | 30.17 | -0.86 |
| Macedonia | 75.56 | 7.13 | 141.71 | 9.34 | 87.55 | 0.94 |
| Madagascar | 204.52 | 6.35 | 427.97 | 6.01 | 109.26 | -0.41 |
| Malawi | 91.27 | 3.51 | 175.69 | 3.55 | 92.5 | -0.83 |
| Malaysia | 309.73 | 5.32 | 1149.33 | 8.10 | 271.07 | 2.03 |
| Maldives | 4.29 | 7.59 | 12.92 | 7.89 | 201.03 | -0.16 |
| Mali | 48.10 | 1.94 | 118.60 | 2.33 | 146.6 | 0.57 |
| Malta | 28.61 | 12.26 | 46.93 | 11.79 | 64.03 | -0.21 |
| Marshall Islands | 0.60 | 5.24 | 2.22 | 10.25 | 269.8 | 2.60 |
| Mauritania | 22.61 | 3.81 | 58.92 | 4.93 | 160.56 | 0.92 |
| Mauritius | 27.32 | 5.73 | 83.28 | 9.83 | 204.84 | 2.17 |
| Mexico | 1535.96 | 5.32 | 5131.09 | 7.85 | 234.06 | 1.47 |
| Moldova | 216.17 | 8.27 | 179.34 | 6.22 | -17.04 | -1.07 |
| Mongolia | 20.75 | 3.26 | 76.14 | 5.04 | 266.99 | 1.74 |
| Montenegro | 25.96 | 7.47 | 39.41 | 8.47 | 51.83 | 0.48 |
| Morocco | 354.59 | 4.21 | 1126.21 | 6.42 | 217.61 | 1.68 |
| Mozambique | 247.93 | 6.04 | 491.01 | 6.43 | 98.05 | 0.04 |
| Myanmar | 1272.23 | 8.60 | 2852.59 | 10.32 | 124.22 | 0.75 |
| Namibia | 20.32 | 4.64 | 38.95 | 4.38 | 91.73 | -0.78 |
| Nepal | 138.26 | 2.47 | 481.95 | 3.82 | 248.58 | 1.78 |
| Netherlands | 1521.03 | 14.60 | 1736.38 | 11.21 | 14.16 | -1.38 |
| New Zealand | 262.96 | 12.94 | 325.49 | 9.30 | 23.78 | -1.49 |
| Nicaragua | 19.89 | 1.74 | 133.01 | 4.67 | 568.77 | 3.82 |
| Niger | 42.76 | 2.45 | 133.14 | 2.80 | 211.4 | 0.29 |
| Nigeria | 849.00 | 3.58 | 2767.26 | 4.95 | 225.94 | 1.31 |
| North Korea | 473.28 | 4.31 | 885.85 | 5.26 | 87.17 | 0.78 |
| Northern Mariana Islands | 0.72 | 4.92 | 1.89 | 7.08 | 163.94 | 1.49 |
| Norway | 486.10 | 15.37 | 516.45 | 12.26 | 6.24 | -0.98 |
| Oman | 12.50 | 3.21 | 49.78 | 4.45 | 298.42 | 1.10 |
| Pakistan | 3417.32 | 10.77 | 14634.67 | 19.65 | 328.25 | 2.39 |
| Palestine | 26.23 | 4.29 | 98.81 | 5.99 | 276.69 | 1.21 |
| Panama | 28.70 | 3.32 | 120.82 | 5.98 | 321.03 | 2.01 |
| Papua New Guinea | 56.60 | 4.51 | 223.63 | 7.13 | 295.11 | 1.89 |
| Paraguay | 35.50 | 2.62 | 181.69 | 6.06 | 411.74 | 3.20 |
| Peru | 175.74 | 2.31 | 988.87 | 6.09 | 462.69 | 4.43 |
| Philippines | 1466.67 | 6.88 | 5499.60 | 11.98 | 274.97 | 2.49 |
| Poland | 2867.55 | 12.04 | 3944.46 | 12.04 | 37.56 | 0.03 |
| Portugal | 500.08 | 7.24 | 685.63 | 6.71 | 37.11 | -0.55 |
| Puerto Rico | 44.33 | 2.26 | 204.23 | 6.66 | 360.66 | 3.15 |
| Qatar | 2.84 | 5.31 | 28.06 | 7.68 | 889.46 | 1.80 |
| Romania | 1331.95 | 9.32 | 1602.98 | 9.96 | 20.35 | 0.26 |
| Russian Federation | 10879.90 | 10.38 | 11487.05 | 9.39 | 5.58 | -0.59 |
| Rwanda | 130.71 | 6.66 | 265.29 | 6.55 | 102.96 | -0.35 |
| Saint Lucia | 1.33 | 2.56 | 10.33 | 9.63 | 677.51 | 4.85 |
| Saint Vincent and the Grenadines | 0.94 | 2.25 | 5.66 | 8.90 | 504.74 | 4.43 |
| Samoa | 3.31 | 6.58 | 6.03 | 7.93 | 82.1 | 0.66 |
| Sao Tome and Principe | 1.56 | 3.86 | 4.44 | 6.51 | 185.31 | 2.01 |
| Saudi Arabia | 73.44 | 2.17 | 485.42 | 4.19 | 560.98 | 2.42 |
| Senegal | 55.45 | 2.86 | 177.28 | 4.00 | 219.7 | 1.14 |
| Serbia | 598.17 | 10.03 | 798.68 | 11.21 | 33.52 | 0.75 |
| Seychelles | 3.37 | 10.71 | 9.44 | 16.80 | 180.11 | 1.79 |
| Sierra Leone | 28.87 | 2.63 | 81.36 | 3.83 | 181.78 | 1.55 |
| Singapore | 104.56 | 7.42 | 259.58 | 7.09 | 148.27 | -0.17 |
| Slovakia | 375.79 | 11.56 | 554.56 | 12.39 | 47.57 | 0.27 |
| Slovenia | 164.66 | 12.04 | 172.93 | 9.32 | 5.02 | -1.12 |
| Solomon Islands | 3.47 | 3.99 | 13.60 | 6.50 | 292.02 | 1.87 |
| Somalia | 101.95 | 5.96 | 282.46 | 6.91 | 177.07 | 0.25 |
| South Africa | 709.81 | 5.05 | 1570.20 | 5.81 | 121.22 | 0.44 |
| South Korea | 512.25 | 2.48 | 2021.82 | 4.91 | 294.69 | 2.42 |
| South Sudan | 81.64 | 6.31 | 145.62 | 6.19 | 78.37 | -0.36 |
| Spain | 2662.19 | 9.83 | 3593.37 | 8.58 | 34.98 | -0.70 |
| Sri Lanka | 265.34 | 3.99 | 795.28 | 5.97 | 199.72 | 1.67 |
| Sudan | 106.46 | 1.91 | 322.04 | 2.79 | 202.5 | 1.51 |
| Suriname | 3.21 | 2.11 | 29.44 | 9.41 | 816.1 | 5.22 |
| Swaziland | 11.50 | 5.59 | 26.92 | 7.01 | 133.98 | 1.00 |
| Sweden | 1092.20 | 15.77 | 808.83 | 9.41 | -25.94 | -2.11 |
| Switzerland | 613.97 | 11.53 | 654.48 | 8.21 | 6.6 | -1.45 |
| Syria | 73.30 | 2.18 | 253.59 | 3.40 | 245.97 | 1.68 |
| Taiwan (Province of China) | 381.47 | 4.24 | 1517.46 | 8.71 | 297.79 | 2.96 |
| Tajikistan | 68.86 | 4.00 | 175.30 | 5.03 | 154.58 | 0.59 |
| Tanzania | 446.11 | 6.49 | 1116.95 | 7.19 | 150.37 | -0.08 |
| Thailand | 1478.95 | 6.15 | 3581.42 | 7.23 | 142.16 | 0.29 |
| The Bahamas | 3.63 | 3.44 | 26.37 | 12.19 | 626.4 | 4.92 |
| The Gambia | 5.13 | 2.45 | 20.74 | 3.69 | 304.51 | 1.66 |
| Timor-Leste | 9.12 | 4.37 | 29.32 | 6.56 | 221.34 | 1.73 |
| Togo | 26.98 | 3.08 | 91.54 | 3.75 | 239.33 | 0.48 |
| Tonga | 1.31 | 4.09 | 3.08 | 6.96 | 134.34 | 2.19 |
| Trinidad and Tobago | 8.28 | 1.67 | 90.65 | 10.30 | 994.44 | 8.12 |
| Tunisia | 93.39 | 3.33 | 307.39 | 4.76 | 229.14 | 1.02 |
| Turkey | 1279.96 | 6.02 | 2754.47 | 5.96 | 115.2 | -0.04 |
| Turkmenistan | 74.56 | 5.42 | 153.06 | 6.30 | 105.27 | 0.78 |
| Uganda | 280.73 | 6.91 | 598.34 | 6.23 | 113.14 | -1.06 |
| Ukraine | 3407.98 | 8.53 | 4308.29 | 11.55 | 26.42 | 0.78 |
| United Arab Emirates | 11.70 | 4.29 | 123.05 | 6.53 | 951.48 | 1.59 |
| United Kingdom | 7479.63 | 17.06 | 7536.92 | 13.35 | 0.77 | -1.23 |
| United States | 19898.55 | 11.85 | 24889.59 | 9.60 | 25.08 | -1.10 |
| Uruguay | 139.64 | 6.97 | 230.03 | 9.09 | 64.73 | 0.85 |
| Uzbekistan | 170.72 | 2.32 | 588.35 | 3.98 | 244.63 | 2.21 |
| Vanuatu | 2.27 | 5.23 | 9.06 | 9.01 | 298.13 | 2.24 |
| Venezuela | 106.25 | 1.59 | 1178.45 | 7.43 | 1009.17 | 5.05 |
| Vietnam | 1124.45 | 4.45 | 3373.42 | 6.19 | 200.01 | 1.17 |
| Virgin Islands, U.S. | 3.65 | 7.08 | 12.35 | 14.58 | 238.46 | 3.33 |
| Yemen | 54.91 | 1.73 | 254.47 | 2.95 | 363.45 | 2.18 |
| Zambia | 175.06 | 9.32 | 351.46 | 8.13 | 100.77 | -1.05 |
| Zimbabwe | 130.38 | 4.95 | 350.91 | 7.50 | 169.15 | 2.08 |

ASIR: age standardized incidence rate; EAPC: estimated annual percentage change.
