# Supplementary material for: Disease Burden and Attributable Risk Factors of Ovarian Cancer From 1990 to 2017: Findings From the Global Burden of Disease Study 2017
Source: Front Public Health. 2021 Sep 17;9:619581. doi: 10.3389/fpubh.2021.619581 (PMC8484795; doi:10.3389/fpubh.2021.619581)
Supplement: Supplementary Table 2 — The top five countries and territories of ovarian cancer incidence, death, or DALYs in 2017. [file Table_2.DOCX]

**Supplementary Table 2 The top five countries and territories of ovarian cancer incidence, death, or DALYs in 2017.**

| **Rank** | **Incidence (95% UI)** | **Deaths (95% UI)** | **DALYs (95% UI)** |
| --- | --- | --- | --- |
| **1** | China  40646.50 (38111.86 - 43093.19) | China  25040.26 (23558.50 - 26505.37) | China  692806.34 (649947.79 - 734597.46) |
| **2** | India  31441.05 (27725.81 - 36329.42) | India  20621.80 (18228.44 - 23704.20) | India  609130.85 (536743.45 - 702242.28) |
| **3** | United States  24889.59 (23480.51 - 26208.47) | United States  16714.53 (15926.81 -17486.83) | United States  360342.41 (340845.55 - 379530.44) |
| **4** | Pakistan  14634.67 (8467.38 - 23659.27) | Russian Federation  7429.15 (7189.92 - 7648.53) | Pakistan  255498.17 (153831.65 - 405140.92) |
| **5** | Russian Federation  11487.05 (10891.42 - 12082.21) | Pakistan  7346.12 (4559.78 - 11652.35) | Russian Federation  192290.36 (185370.73 - 199038.95) |

DALY: disability adjusted life-year; UI: uncertainty interval.
