# Supplementary material for: Disease Burden and Attributable Risk Factors of Ovarian Cancer From 1990 to 2017: Findings From the Global Burden of Disease Study 2017
Source: Front Public Health. 2021 Sep 17;9:619581. doi: 10.3389/fpubh.2021.619581 (PMC8484795; doi:10.3389/fpubh.2021.619581)
Supplement: Supplementary Table 3 — The bottom five countries and territories of ovarian cancer incidence, death, or DALYs in 2017. [file Table_3.DOCX]

**Supplementary Table 3 The bottom five countries and territories of ovarian cancer incidence, death, or DALYs in 2017.**

| **Rank** | **Incidence (95% UI)** | **Deaths (95% UI)** | **DALYs (95% UI)** |
| --- | --- | --- | --- |
| **1** | Kiribati  1.42 (0.96 - 1.95) | Kiribati  0.76 (0.53 - 1.02) | Kiribati  25.86 (17.66 -35.50) |
| **2** | Northern Mariana Islands  1.89 (1.54 - 2.30) | Northern Mariana Islands  0.91 (0.77 - 1.09) | Northern Mariana Islands  27.48 (22.63 - 33.17) |
| **3** | Marshall Islands  2.22 (1.42 - 3.47) | Marshall Islands  0.98 (0.67 - 1.46) | Marshall Islands  35.37 (23.14 - 54.11) |
| **4** | Dominica  2.36 (2.06 - 2.69) | Dominica  1.49 (1.31 - 1.69) | Dominica  37.79 (33.19 - 42.87) |
| **5** | Tonga  3.08 (2.29 - 4.17) | Tonga  1.71 (1.28 - 2.27) | Tonga  47.09 (34.91 - 63.61) |

DALY: disability adjusted life-year; UI: uncertainty interval.
