# Supplementary material for: Disease Burden and Attributable Risk Factors of Ovarian Cancer From 1990 to 2017: Findings From the Global Burden of Disease Study 2017
Source: Front Public Health. 2021 Sep 17;9:619581. doi: 10.3389/fpubh.2021.619581 (PMC8484795; doi:10.3389/fpubh.2021.619581)
Supplement: Supplementary Table 4 — The deaths of ovarian cancer among 195 countries and territories, and their temporal trends from 1990 to 2017. [file Table_4.DOCX]

**Supplementary Table 4 The deaths of ovarian cancer among 195 countries and territories, and their temporal trends from 1990 to 2017.**

| **Countries and territories** | **1990** | | **2017** | | **1990 - 2017** | |
| --- | --- | --- | --- | --- | --- | --- |
|  | **Deaths No.** | **ASDR per 100,000** | **Deaths No.** | **ASDR per 100,000 No.** | **Change in deaths (%)** | **EAPC No. (95% CI)** |
| Afghanistan | 78.49 | 2.28 | 195.38 | 3.02 | 148.93 | 1.22 |
| Albania | 20.78 | 1.74 | 49.21 | 2.38 | 136.8 | 2.15 |
| Algeria | 112.09 | 1.63 | 366.75 | 2.15 | 227.2 | 1.33 |
| American Samoa | 0.85 | 7.14 | 2.85 | 12.85 | 234.55 | 2.66 |
| Andorra | 2.13 | 7.45 | 4.64 | 6.84 | 118.16 | -0.50 |
| Angola | 61.56 | 2.78 | 208.38 | 3.27 | 238.52 | 0.57 |
| Antigua and Barbuda | 0.35 | 1.25 | 3.08 | 5.64 | 767.39 | 5.58 |
| Argentina | 906.04 | 4.98 | 1367.81 | 4.71 | 50.97 | -0.21 |
| Armenia | 75.96 | 4.80 | 113.36 | 4.87 | 49.23 | 0.11 |
| Australia | 681.63 | 6.34 | 1050.77 | 4.92 | 54.16 | -1.09 |
| Austria | 642.66 | 9.05 | 555.72 | 5.69 | -13.53 | -1.86 |
| Azerbaijan | 75.75 | 2.46 | 179.86 | 3.14 | 137.43 | 0.94 |
| Bahrain | 5.04 | 6.22 | 16.02 | 4.31 | 217.75 | -2.56 |
| Bangladesh | 462.74 | 1.97 | 1257.98 | 2.02 | 171.86 | 0.38 |
| Barbados | 2.06 | 1.27 | 14.45 | 5.65 | 602.39 | 6.01 |
| Belarus | 563.89 | 6.95 | 494.37 | 5.35 | -12.33 | -1.08 |
| Belgium | 818.58 | 9.25 | 739.82 | 5.86 | -9.62 | -1.91 |
| Belize | 0.41 | 0.85 | 3.68 | 2.66 | 786.21 | 4.60 |
| Benin | 24.26 | 2.19 | 72.68 | 2.75 | 199.6 | 0.80 |
| Bermuda | 2.57 | 7.15 | 3.74 | 5.33 | 45.76 | -1.14 |
| Bhutan | 3.09 | 2.30 | 8.84 | 2.89 | 186.15 | 0.88 |
| Bolivia | 48.24 | 2.59 | 206.05 | 4.43 | 327.17 | 1.94 |
| Bosnia and Herzegovina | 98.76 | 3.99 | 213.92 | 6.75 | 116.61 | 2.11 |
| Botswana | 11.19 | 3.27 | 34.72 | 4.40 | 210.4 | 1.67 |
| Brazil | 1838.11 | 3.62 | 4673.32 | 3.73 | 154.25 | -0.12 |
| Brunei | 3.75 | 6.70 | 16.23 | 8.95 | 333.35 | 1.88 |
| Bulgaria | 319.93 | 4.81 | 469.98 | 6.53 | 46.9 | 0.98 |
| Burkina Faso | 59.22 | 2.34 | 129.84 | 2.51 | 119.26 | 0.10 |
| Burundi | 67.39 | 5.06 | 90.05 | 4.09 | 33.62 | -1.09 |
| Cambodia | 95.91 | 3.30 | 280.20 | 4.04 | 192.15 | 0.80 |
| Cameroon | 69.70 | 2.74 | 214.29 | 3.36 | 207.42 | 0.67 |
| Canada | 1284.92 | 7.11 | 1900.43 | 5.41 | 47.9 | -1.23 |
| Cape Verde | 2.00 | 1.53 | 5.86 | 2.45 | 193.41 | 1.85 |
| Central African Republic | 20.91 | 3.09 | 39.77 | 3.36 | 90.18 | 0.29 |
| Chad | 29.03 | 1.86 | 64.93 | 2.40 | 123.69 | 1.00 |
| Chile | 257.01 | 4.51 | 495.62 | 3.93 | 92.84 | -0.65 |
| China | 7522.27 | 1.60 | 25040.26 | 2.47 | 232.88 | 1.68 |
| Colombia | 425.55 | 4.27 | 1115.19 | 3.83 | 162.06 | -0.45 |
| Comoros | 6.69 | 5.79 | 15.93 | 6.15 | 138.25 | 0.09 |
| Congo | 25.94 | 4.02 | 69.61 | 5.07 | 168.29 | 0.70 |
| Costa Rica | 20.52 | 2.15 | 97.41 | 3.66 | 374.61 | 1.67 |
| Cote d'Ivoire | 69.52 | 3.17 | 207.87 | 3.85 | 198.99 | 0.70 |
| Croatia | 278.89 | 7.40 | 298.80 | 6.30 | 7.14 | -0.16 |
| Cuba | 59.38 | 1.12 | 343.69 | 3.61 | 478.78 | 4.51 |
| Cyprus | 27.55 | 6.03 | 50.10 | 5.11 | 81.86 | -0.45 |
| Czech Republic | 689.51 | 8.79 | 765.35 | 6.97 | 11 | -1.02 |
| Democratic Republic of the Congo | 256.76 | 2.77 | 583.60 | 3.00 | 127.3 | 0.10 |
| Denmark | 465.64 | 10.90 | 447.62 | 7.52 | -3.87 | -1.86 |
| Djibouti | 5.24 | 6.11 | 19.06 | 6.57 | 263.95 | 0.11 |
| Dominica | 0.30 | 0.79 | 1.49 | 3.24 | 390.9 | 5.33 |
| Dominican Republic | 20.10 | 0.96 | 128.99 | 2.68 | 541.77 | 3.87 |
| Ecuador | 34.34 | 1.12 | 314.22 | 4.06 | 815.05 | 4.86 |
| Egypt | 449.25 | 2.95 | 1175.85 | 3.83 | 161.74 | 0.91 |
| El Salvador | 22.56 | 1.32 | 117.81 | 3.62 | 422.16 | 4.29 |
| Equatorial Guinea | 3.26 | 2.82 | 11.39 | 4.00 | 248.95 | 1.62 |
| Eritrea | 34.12 | 5.66 | 102.03 | 7.06 | 199.03 | 0.58 |
| Estonia | 103.16 | 7.90 | 101.63 | 6.42 | -1.48 | -1.15 |
| Ethiopia | 700.12 | 6.31 | 1270.52 | 5.93 | 81.47 | -0.42 |
| Federated States of Micronesia | 0.83 | 3.19 | 1.90 | 5.02 | 129.58 | 1.80 |
| Fiji | 3.05 | 1.45 | 7.54 | 1.86 | 147.39 | 1.02 |
| Finland | 327.73 | 7.71 | 379.09 | 5.81 | 15.67 | -1.10 |
| France | 3648.03 | 7.60 | 4160.60 | 5.48 | 14.05 | -1.35 |
| Gabon | 11.32 | 3.52 | 23.32 | 4.13 | 106.03 | 0.56 |
| Georgia | 105.01 | 2.85 | 188.89 | 5.86 | 79.88 | 3.60 |
| Germany | 7285.60 | 9.39 | 6308.72 | 6.22 | -13.41 | -1.72 |
| Ghana | 90.32 | 2.45 | 300.38 | 3.25 | 232.57 | 1.01 |
| Greece | 365.18 | 4.51 | 680.18 | 5.70 | 86.26 | 0.79 |
| Greenland | 2.16 | 12.02 | 3.44 | 10.66 | 59.61 | -0.35 |
| Grenada | 0.64 | 1.63 | 5.41 | 7.25 | 749.83 | 6.41 |
| Guam | 1.01 | 2.40 | 5.47 | 6.01 | 442.95 | 4.15 |
| Guatemala | 16.06 | 0.73 | 142.75 | 2.30 | 789.03 | 4.97 |
| Guinea | 49.91 | 2.78 | 99.16 | 3.48 | 98.67 | 0.86 |
| Guinea-Bissau | 5.77 | 2.50 | 13.35 | 3.23 | 131.22 | 1.02 |
| Guyana | 3.24 | 1.47 | 21.13 | 6.21 | 551.94 | 5.51 |
| Haiti | 36.67 | 2.04 | 147.67 | 3.88 | 302.72 | 2.53 |
| Honduras | 31.09 | 2.63 | 192.64 | 5.85 | 519.61 | 3.16 |
| Hungary | 668.02 | 7.82 | 656.62 | 6.09 | -1.71 | -0.89 |
| Iceland | 13.62 | 8.96 | 14.10 | 5.13 | 3.53 | -2.38 |
| India | 5639.67 | 2.28 | 20621.80 | 3.59 | 265.66 | 1.53 |
| Indonesia | 1903.92 | 3.27 | 4765.91 | 4.05 | 150.32 | 0.83 |
| Iran | 242.34 | 1.72 | 1133.00 | 3.12 | 367.53 | 3.09 |
| Iraq | 104.64 | 2.42 | 272.53 | 2.09 | 160.45 | -0.76 |
| Ireland | 211.92 | 9.69 | 294.31 | 7.79 | 38.88 | -0.89 |
| Israel | 200.81 | 7.67 | 326.32 | 5.45 | 62.5 | -1.62 |
| Italy | 3016.28 | 6.08 | 3838.30 | 5.09 | 27.25 | -0.61 |
| Jamaica | 10.42 | 1.11 | 81.77 | 5.39 | 684.72 | 5.64 |
| Japan | 3449.08 | 3.71 | 5268.91 | 3.35 | 52.76 | -0.44 |
| Jordan | 20.09 | 2.60 | 83.35 | 2.78 | 314.89 | -0.01 |
| Kazakhstan | 344.03 | 4.26 | 524.43 | 5.14 | 52.44 | 0.54 |
| Kenya | 136.51 | 2.94 | 399.01 | 3.31 | 192.29 | 0.28 |
| Kiribati | 0.31 | 1.36 | 0.76 | 1.79 | 144.67 | 1.07 |
| Kuwait | 11.42 | 3.96 | 23.99 | 2.00 | 110.08 | -2.08 |
| Kyrgyzstan | 70.43 | 3.87 | 100.88 | 3.79 | 43.22 | 0.41 |
| Laos | 42.27 | 3.51 | 99.07 | 4.11 | 134.37 | 0.60 |
| Latvia | 204.98 | 9.11 | 197.67 | 8.40 | -3.57 | -0.48 |
| Lebanon | 73.65 | 5.72 | 230.86 | 6.95 | 213.47 | 0.58 |
| Lesotho | 17.37 | 3.06 | 36.34 | 5.06 | 109.18 | 2.43 |
| Liberia | 11.34 | 2.08 | 26.52 | 2.57 | 133.89 | 0.88 |
| Libya | 30.62 | 3.25 | 114.59 | 4.76 | 274.21 | 1.30 |
| Lithuania | 306.16 | 11.01 | 296.20 | 8.88 | -3.25 | -0.89 |
| Luxembourg | 31.89 | 9.96 | 39.76 | 7.68 | 24.68 | -1.04 |
| Macedonia | 44.82 | 4.28 | 86.22 | 5.10 | 92.37 | 0.62 |
| Madagascar | 134.41 | 4.74 | 264.93 | 4.35 | 97.11 | -0.50 |
| Malawi | 63.59 | 2.73 | 111.27 | 2.55 | 74.98 | -1.07 |
| Malaysia | 167.27 | 3.28 | 558.47 | 4.22 | 233.86 | 1.38 |
| Maldives | 2.00 | 4.54 | 5.56 | 4.03 | 178.04 | -0.89 |
| Mali | 33.19 | 1.48 | 71.82 | 1.64 | 116.42 | 0.28 |
| Malta | 20.00 | 8.33 | 34.09 | 7.31 | 70.44 | -0.55 |
| Marshall Islands | 0.28 | 3.05 | 0.98 | 5.54 | 248.36 | 2.40 |
| Mauritania | 16.32 | 2.92 | 36.23 | 3.42 | 122.04 | 0.53 |
| Mauritius | 12.87 | 3.01 | 41.44 | 4.56 | 221.85 | 1.65 |
| Mexico | 868.02 | 3.49 | 2758.43 | 4.38 | 217.79 | 0.90 |
| Moldova | 143.59 | 5.30 | 125.09 | 3.91 | -12.88 | -1.17 |
| Mongolia | 13.42 | 2.28 | 45.49 | 3.38 | 238.91 | 1.57 |
| Montenegro | 15.86 | 4.46 | 25.86 | 4.97 | 63 | 0.42 |
| Morocco | 232.58 | 3.00 | 655.56 | 3.93 | 181.86 | 1.10 |
| Mozambique | 168.62 | 4.65 | 313.95 | 4.80 | 86.18 | 0.02 |
| Myanmar | 726.33 | 5.49 | 1555.31 | 5.91 | 114.13 | 0.35 |
| Namibia | 14.62 | 3.54 | 25.92 | 3.13 | 77.26 | -0.97 |
| Nepal | 95.39 | 1.91 | 314.65 | 2.69 | 229.84 | 1.43 |
| Netherlands | 1056.96 | 9.34 | 1273.76 | 7.05 | 20.51 | -1.41 |
| New Zealand | 167.65 | 7.81 | 212.06 | 5.37 | 26.49 | -1.56 |
| Nicaragua | 10.12 | 1.06 | 66.66 | 2.61 | 558.64 | 3.49 |
| Niger | 29.11 | 1.92 | 86.28 | 2.09 | 196.39 | 0.12 |
| Nigeria | 610.25 | 2.79 | 1647.65 | 3.52 | 170 | 0.94 |
| North Korea | 212.83 | 2.00 | 439.84 | 2.44 | 106.66 | 0.82 |
| Northern Mariana Islands | 0.24 | 2.59 | 0.91 | 3.56 | 286.75 | 1.47 |
| Norway | 316.68 | 8.92 | 325.24 | 6.77 | 2.7 | -1.16 |
| Oman | 7.16 | 2.16 | 21.74 | 2.51 | 203.41 | 0.44 |
| Pakistan | 2089.26 | 7.48 | 7346.12 | 12.30 | 251.61 | 1.95 |
| Palestine | 13.50 | 2.57 | 48.72 | 3.56 | 260.81 | 1.35 |
| Panama | 17.30 | 2.21 | 69.87 | 3.43 | 303.97 | 1.44 |
| Papua New Guinea | 26.71 | 2.60 | 96.43 | 3.98 | 261.06 | 1.83 |
| Paraguay | 21.33 | 1.73 | 106.40 | 3.77 | 398.82 | 3.07 |
| Peru | 101.99 | 1.50 | 554.26 | 3.49 | 443.43 | 3.93 |
| Philippines | 669.31 | 3.79 | 2306.39 | 5.58 | 244.59 | 1.83 |
| Poland | 2007.19 | 7.98 | 3046.15 | 8.16 | 51.76 | 0.16 |
| Portugal | 329.73 | 4.29 | 476.41 | 3.77 | 44.48 | -0.77 |
| Puerto Rico | 27.66 | 1.39 | 139.58 | 3.80 | 404.7 | 2.82 |
| Qatar | 1.63 | 3.92 | 13.41 | 4.94 | 724.89 | 1.20 |
| Romania | 819.15 | 5.33 | 1099.23 | 5.82 | 34.19 | 0.41 |
| Russian Federation | 7109.53 | 6.20 | 7429.15 | 5.39 | 4.5 | -0.76 |
| Rwanda | 90.89 | 5.15 | 166.22 | 4.65 | 82.88 | -0.66 |
| Saint Lucia | 0.84 | 1.68 | 6.39 | 5.85 | 663.27 | 4.58 |
| Saint Vincent and the Grenadines | 0.57 | 1.40 | 3.38 | 5.09 | 494.86 | 4.12 |
| Samoa | 1.75 | 3.90 | 3.21 | 4.44 | 83.12 | 0.47 |
| Sao Tome and Principe | 1.00 | 2.70 | 2.49 | 4.23 | 147.76 | 1.72 |
| Saudi Arabia | 42.09 | 1.49 | 197.23 | 2.34 | 368.62 | 1.70 |
| Senegal | 38.59 | 2.21 | 117.47 | 2.94 | 204.39 | 0.98 |
| Serbia | 398.22 | 6.28 | 612.91 | 7.54 | 53.91 | 1.10 |
| Seychelles | 1.76 | 5.67 | 4.47 | 7.79 | 154.09 | 1.17 |
| Sierra Leone | 20.39 | 2.03 | 51.00 | 2.79 | 150.1 | 1.35 |
| Singapore | 63.87 | 4.89 | 131.45 | 3.54 | 105.81 | -1.27 |
| Slovakia | 226.72 | 6.70 | 327.05 | 6.57 | 44.25 | 0.01 |
| Slovenia | 116.46 | 7.92 | 130.68 | 5.85 | 12.21 | -1.26 |
| Solomon Islands | 1.73 | 2.44 | 6.66 | 3.89 | 284.71 | 1.90 |
| Somalia | 69.38 | 4.68 | 187.11 | 5.24 | 169.69 | 0.19 |
| South Africa | 427.95 | 3.37 | 1002.99 | 3.90 | 134.37 | 0.65 |
| South Korea | 284.32 | 1.51 | 1137.33 | 2.50 | 300.01 | 1.63 |
| South Sudan | 55.35 | 4.91 | 89.63 | 4.57 | 61.93 | -0.55 |
| Spain | 1492.24 | 4.95 | 2186.90 | 4.37 | 46.55 | -0.58 |
| Sri Lanka | 136.92 | 2.30 | 398.90 | 2.91 | 191.34 | 1.06 |
| Sudan | 69.04 | 1.40 | 171.64 | 1.82 | 148.6 | 1.09 |
| Suriname | 1.98 | 1.42 | 17.94 | 5.72 | 803.88 | 4.94 |
| Swaziland | 7.36 | 4.17 | 16.97 | 5.04 | 130.58 | 0.92 |
| Sweden | 701.64 | 8.96 | 607.44 | 5.74 | -13.43 | -1.80 |
| Switzerland | 413.16 | 7.00 | 462.81 | 5.00 | 12.02 | -1.40 |
| Syria | 40.59 | 1.38 | 128.88 | 1.87 | 217.54 | 1.32 |
| Taiwan (Province of China) | 162.27 | 2.01 | 623.78 | 3.19 | 284.42 | 1.75 |
| Tajikistan | 44.65 | 2.78 | 96.40 | 3.23 | 115.92 | 0.28 |
| Tanzania | 306.23 | 4.97 | 684.23 | 5.09 | 123.44 | -0.31 |
| Thailand | 732.46 | 3.48 | 1728.82 | 3.24 | 136.03 | -0.52 |
| The Bahamas | 1.96 | 2.10 | 14.22 | 6.78 | 624.23 | 4.60 |
| The Gambia | 3.39 | 1.88 | 13.48 | 2.71 | 298.04 | 1.47 |
| Timor-Leste | 4.82 | 2.89 | 15.67 | 3.79 | 224.93 | 1.16 |
| Togo | 17.40 | 2.34 | 57.11 | 2.68 | 228.1 | 0.28 |
| Tonga | 0.73 | 2.53 | 1.71 | 3.98 | 133.89 | 1.93 |
| Trinidad and Tobago | 4.98 | 1.07 | 56.80 | 6.11 | 1039.57 | 7.79 |
| Tunisia | 57.31 | 2.27 | 174.36 | 2.76 | 204.22 | 0.40 |
| Turkey | 830.71 | 4.19 | 1590.79 | 3.39 | 91.5 | -0.90 |
| Turkmenistan | 37.94 | 3.09 | 78.08 | 3.40 | 105.77 | 0.73 |
| Uganda | 191.03 | 5.37 | 365.89 | 4.54 | 91.54 | -1.26 |
| Ukraine | 2214.23 | 5.05 | 2630.25 | 6.19 | 18.79 | 0.42 |
| United Arab Emirates | 5.65 | 2.94 | 49.01 | 3.90 | 767.16 | 1.15 |
| United Kingdom | 5062.59 | 10.19 | 5093.72 | 7.69 | 0.61 | -1.34 |
| United States | 12692.81 | 6.96 | 16714.53 | 5.70 | 31.69 | -0.99 |
| Uruguay | 102.75 | 4.76 | 161.81 | 5.57 | 57.48 | 0.43 |
| Uzbekistan | 96.32 | 1.41 | 325.06 | 2.44 | 237.49 | 2.32 |
| Vanuatu | 1.06 | 3.05 | 4.45 | 5.21 | 318.06 | 2.28 |
| Venezuela | 55.87 | 0.97 | 638.67 | 4.19 | 1043.16 | 4.71 |
| Vietnam | 669.37 | 2.79 | 1781.20 | 3.35 | 166.1 | 0.61 |
| Virgin Islands, U.S. | 2.31 | 4.78 | 8.40 | 8.61 | 263.86 | 2.77 |
| Yemen | 35.63 | 1.27 | 140.24 | 1.94 | 293.62 | 1.72 |
| Zambia | 112.15 | 7.01 | 214.90 | 5.91 | 91.62 | -1.21 |
| Zimbabwe | 82.97 | 3.59 | 230.90 | 5.54 | 178.31 | 2.25 |

ASDR: age standardized death rate; EAPC: estimated annual percentage change.
