# Supplementary material for: Disease Burden and Attributable Risk Factors of Ovarian Cancer From 1990 to 2017: Findings From the Global Burden of Disease Study 2017
Source: Front Public Health. 2021 Sep 17;9:619581. doi: 10.3389/fpubh.2021.619581 (PMC8484795; doi:10.3389/fpubh.2021.619581)
Supplement: Supplementary Table 5 — The DALYs of ovarian cancer among 195 countries and territories, and their temporal trends from 1990 to 2017. [file Table_5.DOCX]

**Supplementary Table 5 The DALYs of ovarian cancer among 195 countries and territories, and their temporal trends from 1990 to 2017.**

| **Countries and territories** | **1990** | | **2017** | | **1990 - 2017** | |
| --- | --- | --- | --- | --- | --- | --- |
|  | **DALYs No.** | **Age Standardized DALY Rate per 100,000** | **DALYs No.** | **Age Standardized DALY Rate per 100,000 No.** | **Change in DALYs number (%)** | **EAPC No. (95% CI)** |
| Afghanistan | 2547.10 | 70.15 | 7183.59 | 91.16 | 182.03 | 1.14 |
| Albania | 725.47 | 57.59 | 1428.00 | 74.50 | 96.84 | 1.98 |
| Algeria | 3518.39 | 46.86 | 11371.54 | 61.63 | 223.2 | 1.32 |
| American Samoa | 27.48 | 192.99 | 90.67 | 360.17 | 229.94 | 2.77 |
| Andorra | 55.66 | 192.36 | 106.41 | 169.29 | 91.17 | -0.65 |
| Angola | 2118.90 | 80.55 | 6965.81 | 92.01 | 228.75 | 0.43 |
| Antigua and Barbuda | 10.09 | 36.97 | 82.86 | 148.60 | 721.15 | 5.11 |
| Argentina | 23532.77 | 132.88 | 34058.16 | 126.70 | 44.73 | -0.20 |
| Armenia | 2231.31 | 136.35 | 2917.78 | 131.40 | 30.77 | -0.05 |
| Australia | 16577.99 | 164.69 | 21873.00 | 116.05 | 31.94 | -1.45 |
| Austria | 14129.29 | 229.52 | 10930.95 | 133.04 | -22.64 | -2.19 |
| Azerbaijan | 2554.43 | 80.65 | 6239.24 | 104.20 | 144.25 | 1.07 |
| Bahrain | 153.07 | 150.44 | 494.47 | 102.93 | 223.03 | -2.55 |
| Bangladesh | 15304.99 | 57.07 | 40442.86 | 59.26 | 164.25 | 0.46 |
| Barbados | 51.17 | 35.63 | 359.99 | 156.67 | 603.47 | 5.96 |
| Belarus | 15171.58 | 199.12 | 12671.35 | 152.51 | -16.48 | -1.13 |
| Belgium | 17675.79 | 228.57 | 13862.52 | 133.70 | -21.57 | -2.19 |
| Belize | 12.80 | 24.86 | 118.72 | 76.60 | 827.3 | 4.48 |
| Benin | 745.51 | 61.29 | 2310.95 | 76.91 | 209.98 | 0.77 |
| Bermuda | 67.46 | 189.72 | 78.33 | 129.54 | 16.13 | -1.57 |
| Bhutan | 99.74 | 65.06 | 268.65 | 79.71 | 169.35 | 0.76 |
| Bolivia | 1570.43 | 76.03 | 6155.38 | 125.53 | 291.96 | 1.80 |
| Bosnia and Herzegovina | 3103.33 | 121.78 | 5568.62 | 191.50 | 79.44 | 1.75 |
| Botswana | 342.27 | 89.06 | 1037.14 | 115.88 | 203.02 | 1.65 |
| Brazil | 57172.17 | 101.97 | 130630.21 | 103.89 | 128.49 | -0.17 |
| Brunei | 125.87 | 190.30 | 547.85 | 267.50 | 335.24 | 2.01 |
| Bulgaria | 9426.80 | 150.53 | 12093.65 | 196.95 | 28.29 | 0.88 |
| Burkina Faso | 1860.29 | 66.85 | 4166.58 | 70.84 | 123.98 | 0.06 |
| Burundi | 2198.11 | 146.70 | 2961.22 | 114.11 | 34.72 | -1.25 |
| Cambodia | 3239.74 | 99.40 | 8767.13 | 118.23 | 170.61 | 0.67 |
| Cameroon | 2249.22 | 78.53 | 6938.32 | 94.47 | 208.48 | 0.58 |
| Canada | 31008.05 | 183.63 | 40957.10 | 131.94 | 32.09 | -1.49 |
| Cape Verde | 57.52 | 45.05 | 170.21 | 70.21 | 195.93 | 1.75 |
| Central African Republic | 694.76 | 88.68 | 1309.05 | 94.80 | 88.42 | 0.21 |
| Chad | 876.45 | 52.48 | 2092.19 | 66.75 | 138.71 | 0.95 |
| Chile | 7317.91 | 123.68 | 12731.89 | 105.54 | 73.98 | -0.73 |
| China | 262634.83 | 51.21 | 692806.34 | 67.99 | 163.79 | 0.94 |
| Colombia | 13725.62 | 122.81 | 31333.51 | 109.48 | 128.28 | -0.40 |
| Comoros | 223.24 | 171.71 | 498.70 | 178.64 | 123.39 | 0.03 |
| Congo | 815.23 | 114.34 | 2269.34 | 141.77 | 178.37 | 0.60 |
| Costa Rica | 619.23 | 60.54 | 2663.32 | 99.45 | 330.1 | 1.57 |
| Cote d'Ivoire | 2374.04 | 90.13 | 6943.02 | 109.24 | 192.46 | 0.68 |
| Croatia | 7397.35 | 207.04 | 6622.39 | 163.71 | -10.48 | -0.43 |
| Cuba | 1803.22 | 33.63 | 9186.26 | 103.19 | 409.44 | 4.24 |
| Cyprus | 685.29 | 155.97 | 1218.59 | 133.12 | 77.82 | -0.40 |
| Czech Republic | 17714.85 | 243.98 | 17218.66 | 179.97 | -2.8 | -1.33 |
| Democratic Republic of the Congo | 8325.10 | 77.28 | 18224.95 | 81.57 | 118.92 | -0.04 |
| Denmark | 11043.39 | 292.51 | 9070.68 | 175.03 | -17.86 | -2.45 |
| Djibouti | 184.06 | 176.73 | 642.31 | 186.72 | 248.97 | 0.05 |
| Dominica | 8.39 | 24.38 | 37.79 | 91.70 | 350.17 | 5.04 |
| Dominican Republic | 664.48 | 28.03 | 3766.72 | 76.21 | 466.87 | 3.68 |
| Ecuador | 1172.55 | 34.95 | 8992.54 | 112.91 | 666.92 | 4.41 |
| Egypt | 15076.34 | 85.36 | 39242.53 | 109.76 | 160.29 | 0.88 |
| El Salvador | 743.10 | 40.46 | 3498.92 | 107.30 | 370.86 | 4.12 |
| Equatorial Guinea | 105.02 | 80.35 | 371.99 | 110.56 | 254.2 | 1.49 |
| Eritrea | 1144.01 | 162.27 | 3324.38 | 197.33 | 190.59 | 0.45 |
| Estonia | 2611.12 | 216.64 | 2112.45 | 162.26 | -19.1 | -1.58 |
| Ethiopia | 23680.58 | 184.51 | 42584.81 | 170.69 | 79.83 | -0.51 |
| Federated States of Micronesia | 25.91 | 91.36 | 60.63 | 140.40 | 134.02 | 1.70 |
| Fiji | 105.02 | 42.59 | 238.03 | 54.33 | 126.65 | 0.98 |
| Finland | 7473.66 | 196.50 | 7452.85 | 137.77 | -0.28 | -1.39 |
| France | 78278.89 | 189.17 | 77354.02 | 128.03 | -1.18 | -1.63 |
| Gabon | 329.18 | 98.20 | 705.04 | 113.29 | 114.18 | 0.50 |
| Georgia | 3179.87 | 90.31 | 5119.24 | 174.73 | 60.99 | 3.35 |
| Germany | 157572.90 | 233.45 | 122025.16 | 146.24 | -22.56 | -1.93 |
| Ghana | 3006.48 | 71.40 | 9729.93 | 92.86 | 223.63 | 0.94 |
| Greece | 9228.77 | 122.68 | 14112.83 | 146.46 | 52.92 | 0.53 |
| Greenland | 63.28 | 315.09 | 90.94 | 265.76 | 43.71 | -0.56 |
| Grenada | 17.21 | 47.60 | 130.77 | 200.90 | 659.82 | 6.23 |
| Guam | 33.33 | 69.25 | 155.46 | 169.44 | 366.42 | 4.00 |
| Guatemala | 583.13 | 23.55 | 4628.20 | 68.89 | 693.68 | 4.70 |
| Guinea | 1509.12 | 78.73 | 3147.34 | 98.26 | 108.56 | 0.85 |
| Guinea-Bissau | 192.36 | 72.66 | 443.60 | 91.76 | 130.6 | 0.93 |
| Guyana | 108.47 | 43.15 | 671.46 | 184.20 | 519.05 | 5.59 |
| Haiti | 1238.75 | 60.00 | 4775.64 | 107.58 | 285.52 | 2.29 |
| Honduras | 1048.17 | 80.35 | 6132.44 | 172.01 | 485.06 | 3.03 |
| Hungary | 16840.58 | 214.44 | 14749.46 | 159.10 | -12.42 | -1.05 |
| Iceland | 323.60 | 230.85 | 308.44 | 126.16 | -4.69 | -2.56 |
| India | 181959.19 | 64.11 | 609130.85 | 99.86 | 234.76 | 1.46 |
| Indonesia | 65878.66 | 99.50 | 150628.18 | 116.83 | 128.64 | 0.59 |
| Iran | 8311.94 | 50.64 | 34102.47 | 85.48 | 310.28 | 2.83 |
| Iraq | 3566.67 | 74.69 | 9798.96 | 65.31 | 174.74 | -0.76 |
| Ireland | 5120.14 | 254.17 | 6489.63 | 185.08 | 26.75 | -1.30 |
| Israel | 4901.67 | 194.57 | 7177.42 | 132.20 | 46.43 | -1.76 |
| Italy | 71795.22 | 161.83 | 76287.03 | 127.36 | 6.26 | -0.86 |
| Jamaica | 285.11 | 31.53 | 2204.44 | 147.94 | 673.18 | 5.58 |
| Japan | 98324.73 | 111.52 | 112741.26 | 98.51 | 14.66 | -0.48 |
| Jordan | 680.02 | 75.45 | 2621.43 | 74.90 | 285.49 | -0.31 |
| Kazakhstan | 10543.82 | 129.86 | 15756.07 | 152.03 | 49.43 | 0.35 |
| Kenya | 4412.78 | 83.93 | 12981.84 | 94.17 | 194.19 | 0.24 |
| Kiribati | 10.64 | 42.22 | 25.86 | 54.52 | 143.08 | 1.01 |
| Kuwait | 413.90 | 109.05 | 855.27 | 54.62 | 106.64 | -2.08 |
| Kyrgyzstan | 2104.41 | 115.79 | 3258.95 | 114.46 | 54.86 | 0.37 |
| Laos | 1431.36 | 106.65 | 3351.99 | 123.82 | 134.18 | 0.54 |
| Latvia | 5443.52 | 261.01 | 4329.95 | 222.36 | -20.46 | -0.86 |
| Lebanon | 2311.78 | 164.98 | 6529.90 | 187.22 | 182.46 | 0.27 |
| Lesotho | 499.91 | 83.16 | 1021.10 | 132.39 | 104.26 | 2.29 |
| Liberia | 343.72 | 59.47 | 865.14 | 71.59 | 151.7 | 0.76 |
| Libya | 926.58 | 90.05 | 3673.52 | 131.64 | 296.46 | 1.30 |
| Lithuania | 8005.22 | 304.26 | 6625.03 | 237.55 | -17.24 | -1.03 |
| Luxembourg | 721.44 | 249.95 | 822.95 | 180.52 | 14.07 | -1.31 |
| Macedonia | 1426.54 | 132.88 | 2464.91 | 157.17 | 72.79 | 0.55 |
| Madagascar | 4544.21 | 140.66 | 9005.30 | 126.15 | 98.17 | -0.60 |
| Malawi | 2001.00 | 76.98 | 3441.36 | 72.10 | 71.98 | -1.13 |
| Malaysia | 5294.54 | 92.54 | 16748.64 | 117.73 | 216.34 | 1.38 |
| Maldives | 71.29 | 130.99 | 170.27 | 109.12 | 138.84 | -1.03 |
| Mali | 1082.39 | 43.17 | 2348.21 | 46.92 | 116.95 | 0.21 |
| Malta | 487.04 | 207.34 | 711.85 | 175.65 | 46.16 | -0.71 |
| Marshall Islands | 9.88 | 91.46 | 35.37 | 163.77 | 257.84 | 2.29 |
| Mauritania | 480.54 | 81.84 | 1112.16 | 94.90 | 131.44 | 0.50 |
| Mauritius | 410.95 | 89.77 | 1205.46 | 135.10 | 193.34 | 1.69 |
| Mexico | 27966.91 | 100.03 | 84099.70 | 128.32 | 200.71 | 1.01 |
| Moldova | 4474.64 | 169.00 | 3542.63 | 118.64 | -20.83 | -1.36 |
| Mongolia | 426.72 | 68.12 | 1496.23 | 96.52 | 250.64 | 1.34 |
| Montenegro | 458.23 | 129.87 | 672.48 | 140.18 | 46.75 | 0.32 |
| Morocco | 7316.48 | 87.31 | 20109.96 | 113.97 | 174.86 | 1.10 |
| Mozambique | 5651.64 | 135.72 | 10264.06 | 134.65 | 81.61 | -0.19 |
| Myanmar | 24065.75 | 164.67 | 48017.91 | 171.54 | 99.53 | 0.23 |
| Namibia | 436.02 | 100.27 | 737.71 | 84.29 | 69.19 | -1.20 |
| Nepal | 3078.23 | 53.66 | 9405.66 | 74.41 | 205.55 | 1.37 |
| Netherlands | 24096.50 | 236.41 | 24961.23 | 159.42 | 3.59 | -1.85 |
| New Zealand | 4104.31 | 205.72 | 4701.45 | 132.99 | 14.55 | -1.85 |
| Nicaragua | 365.25 | 33.62 | 2096.36 | 76.09 | 473.96 | 3.13 |
| Niger | 963.67 | 54.62 | 2762.70 | 58.12 | 186.68 | 0.01 |
| Nigeria | 17532.58 | 75.27 | 52050.43 | 94.63 | 196.88 | 0.90 |
| North Korea | 7213.01 | 65.09 | 13238.14 | 76.10 | 83.53 | 0.66 |
| Northern Mariana Islands | 9.11 | 70.87 | 27.48 | 93.71 | 201.54 | 1.26 |
| Norway | 7126.80 | 236.80 | 6969.73 | 167.10 | -2.2 | -1.46 |
| Oman | 229.97 | 61.32 | 737.15 | 70.97 | 220.54 | 0.42 |
| Pakistan | 68513.63 | 220.56 | 255498.17 | 360.24 | 272.92 | 1.92 |
| Palestine | 469.41 | 79.97 | 1617.68 | 102.66 | 244.62 | 0.97 |
| Panama | 521.29 | 61.80 | 1912.45 | 94.77 | 266.87 | 1.46 |
| Papua New Guinea | 1010.43 | 82.98 | 3663.19 | 120.81 | 262.54 | 1.62 |
| Paraguay | 673.51 | 50.75 | 3137.70 | 106.25 | 365.87 | 2.87 |
| Peru | 3421.82 | 46.07 | 16214.33 | 101.01 | 373.85 | 3.70 |
| Philippines | 23695.85 | 116.19 | 79028.52 | 174.75 | 233.51 | 1.92 |
| Poland | 57461.80 | 240.63 | 72864.86 | 220.26 | 26.81 | -0.30 |
| Portugal | 8279.31 | 117.30 | 9911.22 | 95.99 | 19.71 | -1.02 |
| Puerto Rico | 744.26 | 38.02 | 3128.82 | 100.21 | 320.39 | 2.71 |
| Qatar | 54.89 | 103.64 | 453.58 | 130.27 | 726.31 | 1.24 |
| Romania | 25227.43 | 171.34 | 27626.42 | 168.02 | 9.51 | -0.05 |
| Russian Federation | 198850.31 | 185.63 | 192290.36 | 153.70 | -3.3 | -0.99 |
| Rwanda | 2965.19 | 149.59 | 5286.35 | 131.52 | 78.28 | -0.82 |
| Saint Lucia | 24.37 | 48.63 | 176.65 | 162.51 | 624.87 | 4.39 |
| Saint Vincent and the Grenadines | 16.44 | 41.26 | 97.73 | 151.83 | 494.56 | 4.09 |
| Samoa | 57.00 | 115.76 | 97.64 | 128.40 | 71.29 | 0.37 |
| Sao Tome and Principe | 31.90 | 81.94 | 82.69 | 126.01 | 159.27 | 1.64 |
| Saudi Arabia | 1354.26 | 41.75 | 7261.93 | 66.90 | 436.23 | 1.77 |
| Senegal | 1205.63 | 62.08 | 3638.46 | 82.31 | 201.79 | 0.96 |
| Serbia | 11833.53 | 189.70 | 14976.15 | 205.63 | 26.56 | 0.60 |
| Seychelles | 52.74 | 174.66 | 133.51 | 230.45 | 153.17 | 1.10 |
| Sierra Leone | 629.91 | 57.72 | 1626.96 | 78.40 | 158.29 | 1.30 |
| Singapore | 1996.84 | 140.61 | 3744.19 | 101.30 | 87.51 | -1.28 |
| Slovakia | 6184.50 | 191.63 | 8124.25 | 179.54 | 31.36 | -0.18 |
| Slovenia | 2968.36 | 213.26 | 2829.59 | 149.63 | -4.67 | -1.51 |
| Solomon Islands | 62.97 | 73.64 | 230.31 | 111.67 | 265.77 | 1.68 |
| Somalia | 2367.54 | 134.10 | 6093.42 | 149.44 | 157.37 | 0.13 |
| South Africa | 13433.73 | 97.80 | 27949.11 | 103.73 | 108.05 | 0.17 |
| South Korea | 9740.86 | 47.06 | 29324.76 | 68.95 | 201.05 | 1.33 |
| South Sudan | 1764.17 | 137.49 | 3065.36 | 129.85 | 73.76 | -0.50 |
| Spain | 37262.38 | 137.09 | 46388.14 | 112.85 | 24.49 | -0.86 |
| Sri Lanka | 4525.65 | 68.90 | 11487.40 | 83.44 | 153.83 | 0.90 |
| Sudan | 2251.14 | 40.68 | 5850.20 | 52.67 | 159.88 | 1.08 |
| Suriname | 61.76 | 40.85 | 518.84 | 162.54 | 740.13 | 4.80 |
| Swaziland | 234.90 | 115.00 | 509.38 | 134.04 | 116.85 | 0.79 |
| Sweden | 15738.11 | 236.10 | 12035.41 | 137.37 | -23.53 | -2.24 |
| Switzerland | 9240.45 | 178.19 | 8869.45 | 113.52 | -4.01 | -1.85 |
| Syria | 1437.73 | 43.56 | 4284.91 | 57.35 | 198.03 | 1.06 |
| Taiwan (Province of China) | 5319.47 | 60.50 | 17956.13 | 97.31 | 237.56 | 1.89 |
| Tajikistan | 1326.47 | 79.33 | 3256.77 | 94.94 | 145.52 | 0.40 |
| Tanzania | 9751.84 | 141.79 | 21951.10 | 144.74 | 125.1 | -0.38 |
| Thailand | 24018.20 | 102.28 | 50585.73 | 96.90 | 110.61 | -0.50 |
| The Bahamas | 63.72 | 62.60 | 433.52 | 195.70 | 580.31 | 4.44 |
| The Gambia | 108.13 | 52.77 | 414.58 | 75.60 | 283.42 | 1.48 |
| Timor-Leste | 173.48 | 84.47 | 476.98 | 109.73 | 174.95 | 1.13 |
| Togo | 586.71 | 67.17 | 1842.31 | 75.88 | 214.01 | 0.21 |
| Tonga | 22.40 | 69.14 | 47.09 | 106.12 | 110.25 | 1.81 |
| Trinidad and Tobago | 151.29 | 31.30 | 1550.18 | 171.17 | 924.67 | 7.62 |
| Tunisia | 1630.58 | 58.31 | 4743.38 | 72.70 | 190.9 | 0.52 |
| Turkey | 25440.19 | 120.17 | 45339.98 | 97.67 | 78.22 | -0.85 |
| Turkmenistan | 1362.61 | 104.08 | 2704.81 | 110.20 | 98.5 | 0.49 |
| Uganda | 6155.74 | 151.13 | 11781.37 | 125.76 | 91.39 | -1.39 |
| Ukraine | 62491.78 | 155.05 | 74165.05 | 196.29 | 18.68 | 0.50 |
| United Arab Emirates | 212.18 | 84.70 | 1919.81 | 110.95 | 804.79 | 1.03 |
| United Kingdom | 115440.71 | 267.77 | 102265.13 | 181.11 | -11.41 | -1.82 |
| United States | 291864.75 | 178.23 | 360342.41 | 137.01 | 23.46 | -1.24 |
| Uruguay | 2587.11 | 130.49 | 3735.39 | 149.63 | 44.38 | 0.38 |
| Uzbekistan | 3177.91 | 44.81 | 10684.36 | 72.06 | 236.21 | 1.96 |
| Vanuatu | 39.15 | 94.27 | 151.45 | 154.11 | 286.86 | 2.06 |
| Venezuela | 1923.00 | 29.87 | 18957.83 | 119.54 | 885.85 | 4.45 |
| Vietnam | 19661.71 | 80.14 | 52656.91 | 95.78 | 167.81 | 0.64 |
| Virgin Islands, U.S. | 67.68 | 128.18 | 204.79 | 230.12 | 202.59 | 2.76 |
| Yemen | 1168.75 | 37.11 | 4728.17 | 56.74 | 304.55 | 1.73 |
| Zambia | 3861.66 | 205.17 | 7126.75 | 168.30 | 84.55 | -1.32 |
| Zimbabwe | 2645.33 | 101.56 | 7326.56 | 155.59 | 176.96 | 2.25 |

DALY: disability adjusted life-year; EAPC: estimated annual percentage change.
